# Lactobacilli Isolated from the Caecum of Healthy Broilers with Antimicrobial Activity are Probiotic Candidates for Controlling *Salmonella*

**DOI:** 10.1007/s12602-026-10980-5

**Published:** 2026-03-19

**Authors:** Schwann Chuwatthanakhajorn, Amy Wedley, Amyleigh Watts, Rachael Slater, Kraig Green, Chris Probert, Witthawat Wiriyarat, Paul Wigley, Barry J. Campbell

**Affiliations:** 1 Infection Biology & Microbiomes, Institute of Infection, Veterinary & Ecological Sciences, University of Liverpool, Liverpool, L69 3GE UK; 2 Faculty of Veterinary Science, Mahidol University, Salaya, Nakhon Pathom, 73170 Thailand; 3 Clinical Infection, Microbiology & Immunology, Institute of Infection, Veterinary and Ecological Sciences, University of Liverpool, Liverpool, L69 7BE UK; 4 Molecular & Clinical Cancer Medicine, Institute of Systems, Molecular and Integrative Biology, University of Liverpool, Liverpool, L69 3GE UK; 5 School of Veterinary Sciences, University of Bristol, Bristol, UK

**Keywords:** Antimicrobial metabolites, Lactic acid bacteria, Lactobacillus, Salmonella, Volatile organic compounds

## Abstract

With the trend shifting towards reducing antibiotic usage, the utilization of lactobacilli as a probiotic is emerging as an alternative, effective method for mitigating *Salmonella* infection in the poultry industry. Here, we investigated the antimicrobial activity of cell-free culture supernatants (CFCS) of three lactobacilli (*Lactobacillus (Lact.) johnsonii*, *Lact. reuteri* and *Lact. crispatus*) isolated from caecal microbiota from mature, healthy broiler chickens, against five *Salmonella enterica* serovars (*Salm.* Typhimurium 4/74, *Salm.* Enteritidis P125109, *Salm.* Enteritidis AviPro^®^, *Salm.* Gallinarum 9 and *Salm*. Gallinarum 287/91). Lactobacilli CFCS demonstrated potent bactericidal activity against all *Salmonella* serovars although the degree of inhibition varied amongst the three strains, where MIC ranged from 8.98 to 18.58 mg/mL, and MBC from 12.62 to 25.54 mg/mL. In support, co-culture assays confirmed all serovars as being completely inhibited by lactobacilli CFCS within 3–6 h. In agar well diffusion assays, *Lact. crispatus* demonstrated highest inhibitory activity, with *Salm.* Gallinarum showed highest susceptibility. Lactobacilli CFCS significantly suppressed *Salmonella* invasion of caecal enterocytes and proinflammatory cytokine release from macrophages. Molecular characterisation of inhibitory moieties within lactobacilli CFCS established they were ≤ 3 kDa, pH dependant, heat tolerant and resistant to proteases. Head-space solid-phase microextraction-gas chromatography/mass spectrometry identified volatile organic short- and medium-chain fatty acids, aldehydes, alcohols, ketones and pyrazines as candidate anti-*Salmonella* post-biotics generated by lactobacilli. In conclusion, lactobacilli isolated from the caecal microbiota of healthy broiler chickens exhibit excellent potential as probiotic candidates to control salmonellosis in poultry and warrant testing in flock intervention studies.

**Supplementary Information:**

The online version contains supplementary material available at 10.1007/s12602-026-10980-5.

## Introduction

Salmonellosis is a significant zoonotic disease affecting humans and animals worldwide. The main causative agent is *Salmonella (Salm.) enterica* subsp. *enterica*, a facultative anaerobic Gram-negative bacterium belonging to Enterobacteriaceae family. The bacterial transmission involves several ecological pathways, including foodborne, waterborne, cross-contamination, and vertical transmission [44]. Human salmonellosis is primarily associated with consuming contaminated water or food, such as meat, poultry, eggs, seafood, fruits, nuts and vegetables [57, 60]. Additionally, cross-contamination from contaminated objects (fomites), mechanical vectors, or the environment can also contribute to the spread of the bacterium [45].

Poultry is a major source of foodborne salmonellosis in humans, commonly transmitted through the consumption of contaminated meat or egg products [29]. Contamination can start at the hatchery (through vertical transmission from parent stock), at the farm (via contaminated feed, water, equipment and transportation) and at slaughterhouses (through cross-contamination within the same flock or between previously contaminated flocks in the production line [88, 89] . Several serovars have been identified in chickens as biological reservoirs, with the most prevalent worldwide being *Salm.* Typhimurium (including monophasic variants) and *Salm.* Enteritidis [19]. Whilst these serovars often cause no symptoms in avian species, they commonly cause acute gastroenteritis-related symptoms, known as non-typhoidal salmonellosis, in humans [69]. The symptoms may include fever, diarrhoea, vomiting, nausea and abdominal cramps, and in severe cases may lead to death [56]. *Salm.* Gallinarum is a notable serovar in chickens, host-adapted to Galliforme birds, causing a systemic infection referred to as “fowl typhoid”, with clinical symptoms, including anorexia, diarrhoea, dehydration, weakness and mortality [84]. This disease often results in high morbidity and acute (or sub-acute) mortality within poultry flocks, reducing performance, egg production and hatchability, thus leading to considerable economic losses in the poultry industry [54]. In light of the growing global concern over use of antibiotics for food production, including their primary use as preventives against animal infection, and the associated rise in anti-microbial resistance [5], there is a significant interest in utilizing lactic acid bacteria as probiotics to control key entero-pathogens in the poultry industry, such as *Clostridioides difficile*, *Escherichia coli*, *Campylobacter*, *Shigella* and *Salmonella* [14, 41, 52, 73, 82]. A key attribute of probiotic lactobacilli is the production of postbiotic bioactive compounds, contributing to enhanced growth performance, improved feed efficiency, mucin production, pathogen suppression, immunomodulation and health promotion in chickens [70, 78]. These functional bioactive compounds provide promising health benefits by improving gut epithelial and immune health and combating harmful enteric pathogens [92]. Several studies have reported the inhibitory effects of lactobacilli on *Salmonella*, attributed to their ability to produce antimicrobial substances [32, 49, 71]. Lactobacilli postbiotics include a diverse range of bioactive molecules and metabolites, including organic acids, such as short-chain fatty acids (SCFAs), bacteriocins and antimicrobial peptides (AMPs), hydrogen peroxide, enzymes, exopolysaccharides, extracellular vesicles and other specialised antimicrobial agents [65]. Their broad spectrum of action and natural origin highlight their potential as an alternative to antibiotic usage to provide effective solutions for controlling infectious disease and as gut health modulators in poultry farming systems [24].

A recent study from our own research group has demonstrated that the transfer of beneficial gut microbiota, using caecal contents from healthy broilers to naïve chicks improves gut health, provides protection against *Salmonella*, and hinders transmission from bird to bird [61]. We identified several lactobacilli from the caecal content transfer material, including three candidate isolates — *Lactobacillus (Lact.) johnsonii*, *Lact. reuteri* (reclassified as *Limosilalactobaciilus reuteri*) [91] and *Lact. crispatus* — which showed ability to be frozen, or freeze dried, and remain viable, as well as demonstrating ability to colonise chicken gut, suggesting strong potential as commercial probiotics for enhancing gut health and to counter enteric pathogens. Thus, the aim of the present study was to evaluate these potential probiotic lactobacilli for their ability to produce antimicrobial metabolites against key five *Salmonella* serovars that significantly impact poultry production. Furthermore, molecular characterisation of identified anti-*Salmonella* post-biotic compounds within cell-free culture supernatants (CFCS) of these lactobacilli would be undertaken utilising Head-space Solid-Phase Microextraction Coupled with Gas Chromatography–Mass Spectrometry (HS-SPME GC-MS).

## Materials and Methods

### Bacterial Isolates and Growth Conditions

All bacteria stocks were stored in Microbank™ bead vials (Pro-Lab Diagnostics; Bromborough, UK) at −80 °C.

#### Lactobacilli

We used three strains of lactobacilli (*Lact. johnsonii* [T37], *Lact. reuteri* [T42] and *Lact. crispatus* [T43]) identified from caecal content collected from healthy 7-week-old Ross 308 broilers as described previously [61] to produce caecal microbiome transplant material; see Supplementary Information file S1. Broiler chicks were hatched and housed in the University of Liverpool high biosecurity facility. All work was carried out in accordance with UK legislation governing use of experimental animals under project license number PPL 40/3652 and was reviewed and approved by the University of Liverpool (Liverpool, UK) ethical review process.

These lactobacilli were selected as they showed ability to be frozen or freeze dried and remain viable, as well as demonstrating ability to colonise in the chicken. Lactobacilli were cultured in de Man, Rogosa and Sharpe (MRS) agar (Neogen NCM0079A - Trafalgar Scientific; Heywood, UK). The cultures were incubated under microaerobic conditions (80% N_2_, 12% CO_2_, 5% O_2_, and 3% H_2_) at 41 °C for 18–24 h. After selecting a single colony, the bacteria were placed in 10 mL of MRS broth and incubated overnight at 41 °C under microaerobic conditions. For growth curve analysis, a single colony of each strain was inoculated into 10 mL of MRS broth and incubated at 41 °C for 24 h, under microaerobic conditions. The following day, 0.1 mL of the overnight culture was transferred to 9.9 mL of fresh MRS broth and incubated at 41 °C under microaerobic conditions. At hourly intervals, 0.2 mL samples were collected, and bacterial concentrations were determined by overnight culture on LB agar using the Miles and Misra technique [50].

#### *Salmonella* Serovars

We also included 5 key serovars of *Salmonella enterica* in the study: *Salm*. Gallinarum 287/81, a field isolate obtained from a chicken in Brazil in 1991 [77]; *Salm*. Gallinarum 9, isolated from a diseased chicken in the UK in 1954 [74]; *Salm*. Enteritidis P125109, a phage type 4 isolate from a UK food poisoning outbreak associated with consumption of infected eggs [77] and *Salm*. Typhimurium 4/74, an ST19 isolate associated with diarrhoea in calves isolated in the UK in 1974, the progenitor of the laboratory strain SL1344 [33]. These strains were gifts from Professor Paul Barrow (Institute for Animal Health, University of Nottingham, UK). *Salm*. Enteritidis Avipro^®^ (AviPro tadE), a strain used in the Elanco AviPro vaccine formerly produced by Lohmann Animal Health (now Elanco; Cuxhaven, Germany), was the kind gift of Dr Daniel Windhorst (formerly of Lohmann Animal Health). All strains are well-characterised for virulence and the ability to colonise the chicken gut [16, 35, 85]. To culture *Salmonella* serovars, Luria-Bertani (LB) agar (L3022 - Sigma-Aldrich; Poole, UK) was used and plates incubated under aerobic conditions, at 37 °C for 18–24 h. After selecting a single colony, the colony was transferred to 10 mL of LB broth and incubated overnight at 37 °C within an orbital shaker (Sanyo Gallenkamp Plc; Cambridge, UK) at 150 rpm under aerobic conditions. For growth curve analysis, a single colony was inoculated into 10 mL of relevant broth and incubated at 37 °C for 18–24 h under aerobic conditions. The following day, 0.1 mL of the overnight culture was transferred to 9.9 mL of fresh LB broth and incubated at 37 °C with shaking at 150 rpm under aerobic conditions. At half hour intervals, 0.2 mL samples were collected to measure optical density at 600 nm (OD_600nm_) using a Multiskan FC Microplate Photometer (Fisher Scientific; Loughborough, UK), and bacteria enumerated at each time point by overnight culture on LB agar.

### Preparation of Lactobacilli Cell-free Culture Supernatants (CFCS)

A single colony of each *Lactobacillus* spp. was inoculated into 10 mL of MRS broth and incubated overnight at 41 °C, under microaerobic conditions. The following day, overnight cultures were centrifuged at 2,750 × *g* for 5 min. Each culture supernatant was filtered using a low protein adsorbing 0.22 μm Minisart^®^ polyethersulfone (PES) membrane filter (Sartorius AG, Göttingen, Germany). The pH was measured, and the filtrates were stored at − 20 °C until testing.

#### Lyophilization of Lactobacilli CFCS

Aliquots (10 mL) of CFCS were placed in 25 mL universal tubes overnight at −20 °C freezer, then brought further down in temperature to −80 °C for 1 h, before being lyophilised in a Modulyo EF4 freeze dryer (Edwards High Vacuum; Burgess Hill, UK) operated at − 40 °C, at 8 mbar for 48 h. Lyophilised CFCS were weighed, reconstituted to 1 mL using sterile, deionised water, for testing at 10X strength in assays.

### Minimum Inhibitory Concentration (MIC) and Minimum Bactericidal Concentration (MBC) Determinations

The MIC of Lactobacilli CFCS’s were determined in LB broth, following the broth microdilution protocol for antibacterial testing [6]. Briefly, 50 µL of LB broth was added to wells 2–12 of each row across a sterile 96-well plate. Lyophilized CFCS was weighed and reconstituted in 1 mL of sterile water. Subsequently, 100 µL of the reconstituted 10X CFCS was added to the first well of the plate. A two-fold serial dilution was performed by transferring 50 µL from the first well to the next well containing 50 µL of LB broth and mixed by gentle aspiration. This procedure was repeated sequentially through to the eleventh well. A volume of 50 µL was discarded from the eleventh well to ensure equal volumes across all wells. The final (twelfth) well contained 50 µL of LB broth without CFCS, serving as a positive control for *Salmonella* growth, with 50 µL of *Salmonella* suspension (1 × 10⁶ CFU/mL, determined from a standard growth curve correlating OD_600nm_ values measured using a GenQuant Pro spectrophotometer (GE Healthcare/Amersham Biosciences; Chalfont St. Giles, UK) with viable bacteria counts of the same samples following overnight growth on LB agar, established for each serovar) added to all wells on the plate; final total volume of 100 µL per well. Plates were then incubated at 37 °C for 24 h under aerobic conditions. The lowest inhibiting concentration was determined from measurements made at OD_600nm_. To identify the MBC, 20 µL of suspensions (bacteria–treatment mixtures collected from the MIC assay wells) were cultured on LB agar plate, incubated at 37 °C for 24 h. The MBC was the concentration where growth on the plate was no longer detected [6]. These experiments were conducted in triplicate for each CFCS.

### Agar Well Diffusion Assays

To prepare the *Salmonella* loaded agar plates, 1 mL of *Salmonella* (at a concentration of 10^6^ CFU/mL) was mixed with 19 mL of LB agar (with agar at a temperature of 45 °C at time of mixing with bacteria), and plates incubated overnight at 37 °C under aerobic conditions. Lyophilised CFCS were each reconstituted in 1 mL sterile water. Agar wells were aseptically created using a 0.6 mm sterile stainless-steel corkborer on the agar plate, followed by addition of 100 µL of lyophilised, reconstituted 10X CFCS to each well. Regarding experimental controls, MRS broth was used as a negative control, and Oxoid 10 µg gentamicin antimicrobial susceptibility discs (Scientific Laboratory Supplies; Nottingham, UK) used as a positive inhibition control, based on a previously published method [34]. Use of a commercially defined amount of a clinically relevant macrolide antibiotic, ensured enhanced reproducibility and interpretability of inhibition zone data both within and between assays. After overnight incubation at 37 °C under aerobic conditions, the inhibition zones were measured and recorded.

### Lactobacilli CFCS-*Salmonella* Co-culture (Time-kill) Assays

To examine the time-dependent interaction between lactobacilli CFCS and *Salmonella*, a co-culture assay was performed assay based on the method of Mariam and colleagues [48]. One mL of 10X CFCS from each lactobacilli strain was combined with 1 mL of *Salmonella* culture (10^8^ CFU/mL) in LB broth. Samples were taken at the start of the culture (time 0) and further samples were taken following 3, 6 and 24 h of co-culture. The number of viable *Salmonella* were quantified following plating of samples to LB agar using the Miles and Misra method. Following overnight incubation at 37 °C, CFU were enumerated.

### *Salmonella*-chick Caecal Epithelial Cell Invasion Assays

The 8E11 cell-line, a model of chicken intestinal/caecal enterocytes (CHIC clone 8E11), was obtained from Tentamus Pharma & Med Deutschland GmbH (Bad Kissingen, Germany). Cells were initially grown for 2–3 days in Corning ULA 75cm^2^ tissue culture flasks (AppletonWoods; Birmingham, UK) in DMEM/F-12 medium supplemented with 10% v/v foetal bovine serum (FBS), 10 U/mL penicillin, 10 mg/mL streptomycin and 2 mM L-glutamine (Sigma-Aldrich; Poole, UK), in a humidified incubator at 37 °C, an atmosphere of 5% CO_2_. Confluent cells were detached using 0.05% w/v Trypsin-EDTA (Gibco; Paisley, UK), counted using a TC10 automated cell counter (Bio-Rad; Hemel Hempstead, UK) and seeded to 24 well plates (Greiner CELLSTAR flat bottom; Sigma-Aldrich, Poole, UK) at 1 × 10^5^ cells/well in 1 mL culture medium, then incubated at 37 °C, 5% CO_2_ for 2–3 days until fully confluent. Twenty-four hours prior to infection, cell monolayers were washed three times with sterile PBS, followed by addition of 1 mL of antibiotic-free culture medium per well. One hour before infection, 100 µL of 10X CFCS was added to each well of treatment group. To determine the number of host cells for multiplicity of infection (MOI), additional control monolayers grown on separate culture plates were detached using 0.05% w/v Trypsin-EDTA at 37 °C in the presence of 5% CO_2_ in a humidified incubator for 5 min and a single cell suspension in culture medium enumerated using a TC10 automated cell counter. On average, 1 × 10^6^ chicken caecal epithelial cells were present per well after 60 h of incubation (initially seeded at 1 × 10^5^ cells per well). A total of 1 × 10^7^ CFU of *Salmonella* serovars were added per well, i.e. MOI 10. Infected cells were incubated for 1 h at 37 °C, under 5% CO_2_ conditions in a humidified incubator. Following infection, the cells were washed 3 times with sterile PBS and 1 mL of culture medium supplemented with 100 µg/mL gentamicin (Sigma-Aldrich; Poole, UK) was added, and cells incubated for a further hour. The cells were washed twice, then lysed with 1 mL of 1% v/v Triton X-100 in deionised water for 5 min. Invasive *Salmonella* released from infected cells were plated to LB agar, and following overnight culture at 37 °C, CFU were counted.

### Epithelial Cell Cytotoxicity Assay

The impact of lactobacilli CFCS on 8E11 epithelial cell integrity was assessed using the lactate dehydrogenase (LDH) Cytotoxicity Detection Kit (Roche; Mannheim, Germany), following the manufacturer protocol. Cell culture medium was collected post-treatment for assay. Post-treatment with lactobacilli CFCS, cell culture medium was collected for LDH assay. Total maximum releasable intracellular LDH was determined in control (untreated/uninfected) cells, which were washed twice with sterile PBS, then lysed with 1 mL of 1% v/v Triton X-100 for 5 min. Samples (100 µL) were added to wells in a 96-well plate, including fresh cell culture medium as a background control, and the LDH assay reaction conducted at room temperature for 30 min. Absorbance was measured at optical density (OD) 490 nm using a Multiskan FC Microplate Photometer (Fisher Scientific, Loughborough, UK).

### Effect of Lactobacilli CFCS on *Salmonella-*induced Pro-inflammatory Cytokine Release from Macrophages

We investigated the effect of lactobacilli CFCS on pro-inflammatory responses of antigen-presenting cells to *Salmonella* infection and bacterial lipopolysaccharide (LPS).

#### Macrophage Cell Culture

The murine macrophage cell-line J774.A1 was obtained from European Collection of Animal cell Culture (ECACC 85011428 - Public Health Laboratory Service; Wiltshire, UK) was initially grown to confluency in T25 flasks in RPMI 1640 medium supplemented with 10% v/v FBS, 10 U/mL penicillin and 10 mg/mL streptomycin. Macrophages were then split to 24-well plates (Greiner CELLSTAR flat bottom; Sigma-Aldrich UK) at 5 × 10^5^ cells/well in 1 mL culture medium. Cells were incubated at 37 °C, at 5% CO₂ for 24 h. Prior to infection, the cells were washed twice with sterile PBS and antibiotic-free cell culture medium added to each well. One hour before infection, 100 µL of CFCS was added to the treatment group wells. Then, 100 µL of *Salm.* Typhimurium 4/74 (MOI 10) added for 2 h, or 50 ng/mL *Salm.* Typhimurium LPS (437650; Sigma-Aldrich) added for 1 h, and incubated at 37 °C.

#### Enzyme-linked Immunosorbent Assay (ELISA)

Release of pro-inflammatory TNF from the macrophage infection model was detected using the murine TNF Standard TMB ELISA Development Kit (PeproTech; London, UK) according to the manufacturer’s protocol. Absorbance values were measured with a Tecan Sunrise ELISA plate reader (Tecan Trading AG; Männedorf, Switzerland) at OD_450nm_. TNF levels in the samples were quantified against as a recombinant murine TNF standard calibration curve provided by the manufacturer. All samples were assayed in triplicate.

### Characterisation of the Anti-*Salmonella* Activity within Lactobacilli CFCS

To identify the size, and to isolate the inhibitory fractions, of active compounds/metabolites within lactobacilli CFCS, molecular weight cut-off ultrafiltration analysis and size exclusion gel chromatography was performed.

#### Molecular Weight Cut-off Analysis Using ultrafiltration

The molecular size of antimicrobial compounds in lactobacilli CFCS was determined using Amicon^®^ Ultra-0.5 centrifugal filter devices with molecular weight cut-offs of 30, 10 and 3 kDa (Merck Millipore Ltd.; Cork, Ireland). Briefly, 500 µL of 10X lactobacilli CFCS was added to the ultrafiltration devices and centrifuged at 14,000 x *g* for 10 min (for 30 kDa and 10 kDa devices) or 30 min (3 kDa device). Flow-through was collected and retained solutes were recovered by reverse centrifugation (1,000 x *g*, for 2 min). Each was made up with sterile water to a final volume of 500 µL. Both the flow-through and retained concentrate samples were tested for antimicrobial activity against *Salm*. Typhimurium 4/74 using the agar well diffusion assay and compared to a pre-ultrafiltration 10X CFCS for each lactobacilli strain.

#### Sephadex G25 Size Exclusion Gel Chromatography

Lyophilised CFCS (10X) from *Lactobacillus* strains were resuspended in a volume of 1 mL and fractionated into 15 fractions (1 mL each) using Sephadex G25 gel filtration chromatography PD10 columns (GE Healthcare limited; Buckinghamshire, UK). Bovine serum albumin (BSA) and phenol red were used as standards to evaluate the void (V_o_) and small molecule (V_s_) volumes. Each eluted fraction of lactobacilli CFCS was tested for its antimicrobial activity against *Salm*. Typhimurium 4/74 using the agar well diffusion assay. In parallel gel filtration experiments identified inhibitory CFCS fractions were pooled, lyophilized and resuspended in ultrapure water to a final volume of 1 mL for further analysis using GC-MS. All experiments were performed using *N* = 3 CFCS, and fractions assayed in triplicate for anti-microbial activity.

### Impact of Physical and Chemical Treatments on Anti-Microbial Activity of Lactobacilli CFCS

#### Impact of Altered pH on the Antimicrobial Activity of Lactobacilli CFCS

To evaluate the importance of postbiotic pH on the antimicrobial activity of lactobacilli CFCS towards *Salmonella*, studies were performed using CFCS neutralised to pH 7. Briefly, each lactobacilli CFCS was carefully adjusted to pH 7 using 1 M NaOH, monitored with a calibrated HI 2210 pH meter (Hanna Instruments; Woonsocket RI, USA). Aliquots (10 mL) of the pH-adjusted (neutralised) CFCS were subsequently frozen at − 80 °C for 24 h and lyophilised, then reconstituted in 1 mL sterile deionised water (10X CFCS). The antimicrobial activity of the pH-adjusted (neutralised) 10X CFCS against *Salm*. Typhimurium 4/74 was evaluated using the agar-well diffusion assay, compared to standard 10X lactobacilli CFCS. A gentamicin antibiotic disc (10 µg) was included as a positive inhibition control for the assay. Lyophilised, reconstituted (10X) MRS broth was included as a negative control.

#### Heat Tolerance of the Antimicrobial Metabolites within Lactobacilli CFCS

To determine the effect of temperature on the stability of antimicrobial compounds within lactobacilli CFCS, assays were performed based on methods previously described (Lin and Pan, 2019). Briefly, overnight cultures of lactobacilli CFCS were subjected to heat treatment in a water bath set at 40 °C, 60 °C, 80 °C and 95 °C for 30 min, compared to CFCS held at room temperature. In addition, the effect of heat-induced denaturation on antimicrobial metabolites was further examined by autoclave of the lactobacilli CFCS, i.e. at high pressure, saturated steam heat (121 °C, for 20 min) in Astell autoclave (ASB280BT65 - Astell Scientific Ltd; Sidcup, UK). After cooling to room temperature, all experimental samples were aliquoted (10 mL samples), stored at − 80 °C for 24 h and subsequently lyophilised. The antimicrobial activity of the lyophilised CFCS, reconstituted in 1 mL sterile deionised water, was then evaluated for activity against *Salm.* Typhimurium 4/74 using the agar-well diffusion assay. Room temperature lactobacilli CFCS and gentamicin discs were included as positive controls. MRS broth was included as a negative control.

#### Effect of Proteolytic Digestion on the Anti-*Salmonella* Activity of Metabolites within Lactobacilli CFCS

To evaluate if the nature of the postbiotic anti-*Salmonella* activity of lactobacilli CFCS was proteinaceous, the effect of proteolytic enzymes was evaluated, based on the protocol described by Ren and colleagues [67]. Pepsin A [EC 3.4.23.1] (P7000; Sigma-Aldrich UK) and papain [EC 3.4.22.2] (1.07144; Millipore/Merck, Gillingham, UK) were selected based on their activity across an acidic pH range (pH 2–4 for pepsin, and pH 5–7 for papain). Each enzyme was added to lactobacilli CFCS at 1 mg/mL and incubated at 37 °C for 3 h. The enzymes were then inactivated by heating the samples at 90 °C for 15 min. To ensure the validity of the results, additional controls were included (e.g. lactobacilli CFCS treated with enzymes preheated at 95 °C for 15 min prior to enzyme addition, untreated CFCS, heated lactobacilli CFCS without enzyme treatment, and unheated lactobacilli CFCS). The samples were subsequently lyophilised, reconstituted in 1mL sterile deionised water and tested for anti-*Salmonella* activity using the agar-well diffusion assay.

#### Crude Bacteriocin/small Anti-microbial Peptide Activity Assessment of Lactobacilli CFCS

To investigate bacteriocin/anti-microbial peptide (AMP) production in *Lact. johnsonii*, *Lact. reuteri* and *Lact. crispatus*, lactobacilli CFCS was subjected to partial purification using ethyl acetate, based on previously established protocols [42, 87]. Ethyl acetate was added at a ratio 3:2 to the lactobacilli CFCS, thoroughly mixed on a shaker at 200 rpm for 2 h, and allowed to separate on standing for 30 min. The separated components, including the upper ethyl acetate ‘extraction’ phase that contains bacteriocins/AMPs, an emulsion layer (if present), and the lower ‘retained’ aqueous phase fractions, were collected into individual tubes. The organic solvent from the collected upper ‘extraction’ phase was then removed by evaporation in a fume hood, followed by reconstitution with 1/10 of its volume using 0.1X sterile PBS, while the lower ‘retained’ aqueous fraction was collected, freeze-dried and reconstituted to 1/10 of its original volume. The emulsion phase was collected, left in the fume hood to allow ethyl acetate evaporation, then freeze-dried and reconstituted to 1/10 of its original volume. The antimicrobial activity of both the crude ‘extraction’ and the ‘retained’ fractions from each *Lactobacillus* strain was subsequently assessed using the agar-well diffusion assay, as previously described. All experiments were conducted in triplicate.

### Identification of Antimicrobial Volatile Organic Compounds within Lactobacilli CFCS

To identify volatile organic compounds (VOCs), carbon-based molecules/metabolites (e.g. SCFAs, alcohols, etc.) that can be emitted as vapours from biological samples [62], within the lactobacilli CFCS that might be responsible for inhibiting growth of *Salmonella*, an accurate and reliable biological sample extraction method was employed followed by detection using Headspace Solid Phase Micro-Extraction Gas Chromatography/Mass Spectrometry (GC/MS) [22, 23].

Overnight microaerobic MRS broth cultures of lactobacilli CFCS were prepared, lyophilised and reconstituted in 1 mL sterile ultrapure water (18 Ω.cm). Samples were aliquoted within a biosafety containment cabinet into 10 mL clear glass, crimp headspace vials (VI-01–10-RE/22.5 × 46 mm: Chromatography Direct; Wellingborough, UK), with three independent overnight MRS broth culture replicates for each treatment. MRS culture broth alone was used as an experimental control and ambient air samples collected in head-space vials within the hood represented a background VOC control for the HS-SPME-GC/MS analysis. In addition, 10X lactobacilli CFCS were each fractionated using PD10 Sephadex G25^®^ gel filtration chromatography and fractions exhibiting antimicrobial activity against *Salm* Typhimurium 4/74 were pooled, lyophilised, resuspended with ultrapure water to a volume of 1 mL and aliquoted to head-space vials for VOC analysis.

All samples in headspace vials were loaded to a Combi PAL autosampler (CTC Analytics AG; Zwingen, Switzerland) supporting a Perkin Elmer Clarus 500 GC/MS quadrupole bench-top system (Perkin Elmer; Beaconsfield, UK) with a Zebron ZB-624 GC column (i.d. 0.25 mm, length 60 m, film thickness 1.4 μm (Phenomenex, Macclesfield, UK) and 99.996% pure helium carrier gas (BOC; Sheffield, UK). Samples were each heated at 60 °C for 30 min and volatiles extracted from the vial headspace and adsorbed onto the SPME fibre for 20 min, at 60 °C. The SPME fibre coating used was Divinylbenzene/Carboxen/Polydimethylsiloxane (DVB/CAR/PDMS) with needle size 23 gauge (StableFlex; Sigma-Aldrich UK). The fibre was introduced for into the GC component for desorption at 220 °C for 5 min [1]. A background blank headspace vial was run at the start and during the sequence run to check for contaminants in laboratory air when sampling. All GC/MS selected peaks were identified using the National Institute of Standards and Technology (NIST) Mass Spectral library (NIST v.17, 2019) with minimum match factor of ≥ 800 and a probability of ≥ 60%. A batch report was generated from the Automated Mass Spectral Deconvolution and Identification System (AMDIS; v. 2.73, 2017) followed by generation of a spreadsheet using R package Metab [2]d Software (v. 4.1.2, R Foundation for Statistical Computing; Vienna, Austria).

### Statistics

Statistical analyses of data were performed with GraphPad Prism software version 9.5.1 for Windows (San Diego, CA, USA). Data normality was evaluated prior to analysis using the D’Agostino-Pearson omnibus normality test. For data following a normal distribution, one-way ANOVA was performed with a significance level set at *p* < 0.05, followed by Tukey’s post-hoc test for pairwise comparisons. For the GC/MS analysis, compounds selected for further evaluation were required to be present in at least 2 out of 3 biological replicates for a given sample condition. Missing values were imputed with one-fifth of the minimum positive value, and the raw data were normalised using logarithmic transformation. Principal component analysis (PCA), box and whisker plots (constructed to visually represent the spread of data (range) and heat-map generation were performed using the MetaboAnalyst tool v. 6.0 [58]. Benjamini-Hochberg to control false discovery rate (FDR) was applied to adjust *p*-values for multiple comparisons. PCA was conducted on samples to provide a visual representation of the variation between its principal components (the underlying structure of the data). Differences were determined using one-way ANOVA followed by Tukey’s post hoc test, with *p* < 0.05 considered significant.

## Results

### Lactobacilli CFCS Demonstrate Inhibitory Activity Against *Salmonella* Serovars

To assess the impact of lactobacilli CFCS on anti-*Salmonella* activity, MIC and MBC were evaluated (see Table 1). The results revealed varying degrees of antibacterial activity among the three strains of lactobacilli CFCS against different *Salmonella* strains. Minimum inhibitory concentrations (MIC) ranged from 8.98 ± 3.56 to 18.58 ± 5.77 mg/mL. Individually, *Lact. reuteri* CFCS exhibited the highest inhibitory effect against all *Salmonella* serovars, with concentrations ranging from 8.98 ± 3.56 to 11.16 ± 3.49 mg/mL, while *Lact. crispatus* and *Lact. johnsonii* CFCS showed inhibitory effects ranging from 9.33 ± 0.61 to 15.75 ± 3.66 mg/mL and 10.56 ± 1.72 to 18.58 ± 5.77 mg/mL, respectively. The minimum bactericidal activity (MBC) of CFCS ranged from 14.04 ± 3.82 to 25.54 ± 9.88 mg/mL for *Lact. johnsonii*, 12.62 ± 2.10 to 17.95 ± 7.13 mg/mL for *Lact. reuteri*, and 15.76 ± 3.64 to 21.55 ± 7.49 mg/mL for *Lact. crispatus*.

**Table 1:** Inhibitory activity of lactobacilli CFCS on *Salmonella* serovar growth

Salmonella serovar	MIC/MBC	Lact. johnsonii (mg/mL)	Lact. reuteri (mg/mL)	Lact. crispatus (mg/mL)
*Salm*. Typhimurium 4/74	MIC	18.58 ± 5.77	11.16 ± 3.49	15.75 ± 3.66
	MBC	18.58 ± 5.77	17.95 ± 7.13	18.66 ± 1.22
*Slma*. Enteritidis P125109	MIC	14.04 ± 3.82	11.16 ± 3.49	14.30 ± 5.06
	MBC	18.58 ± 5.77	17.95 ± 7.13	21.55 ± 7.49
*Salm*. Enteritidis AviPro^®^	MIC	14.04 ± 3.82	11.16 ± 3.49	14.30 ± 5.06
	MBC	21.13 ± 3.45	17.95 ± 7.13	15.76 ± 3.64
*Salm*. Gallinarum 9	MIC	14.04 ± 3.82	11.16 ± 3.49	11.40 ± 5.01
	MBC	14.04 ± 3.82	12.62 ± 2.10	15.76 ± 3.64
*Salm*. Gallinarum 287/91	MIC	10.56 ± 1.72	8.98 ± 3.56	9.33 ± 0.61
	MBC	25.54 ± 9.88	16.50 ± 8.15	18.66 ± 1.22

Footnote: Mean (± SD) dry weight of CFCS after lyophilization from 10 mL overnight culture for *Lact. johnsonii* (T37, 132.76 ± 30.39 mg)

*Lact. reuteri* (T42, 124.37 ± 17.83 mg) and *Lact. crispatus* (T43, 149.47 ± 16.93 mg). Inhibitory activity data reported as mean ± SEM of triplicate experiments. MIC, minimum inhibitory concentration; MBC, minimum bactericidal concentration

### Anti-*Salmonella* Activity of Lactobacilli CFCS as Shown by Agar well Diffusion Assays

The antimicrobial activity of *Lact. johnsonii*, *Lact. reuteri* and *Lact. crispatus* CFCS against the five *Salmonella* serovars is presented in Table 2. The results revealed a consistently high inhibitory effect of the CFCS from all three lactobacilli against various *Salmonella* serovars, with minimal variation in inhibition levels. Regarding assessment of the CFCS-induced inhibition zones for *Salm*. Typhimurium 4/74, *Lact. crispatus* demonstrated the highest inhibitory effect, followed by *Lact. johnsonii* and *Lact. reuteri*. The mean (± SD) inhibition zones observed were 16.11 ± 1.83 mm, 15.77 ± 0.62 mm and 15 ± 1.15 mm, respectively. For *Salm*. Enteritidis P125109 and *Salm*. Enteritidis AviPro^®^, a comparable level of susceptibility was discerned among the strains. In the case of *Salm*. Enteritidis P125109, *L. crispatus* achieved the largest inhibition zone, followed by *Lact. johnsonii* and *Lact. reuteri*, with average inhibition zones of 15.77 ± 1.22 mm, 15.66 ± 0.47 mm, and 15.44 ± 0.49 mm, respectively. Similarly, for *Salm.* Enteritidis AviPro^®^, the highest inhibition zone was observed with *Lact. crispatus*, followed by *Lact. johnsonii* and *Lact. reuteri*, with average inhibitions of 16.55 ± 1.83 mm, 14.77 ± 1.03 mm, and 14.44 ± 1.83 mm, respectively. *Salm*. Gallinarum demonstrated the strongest susceptibility to lactobacilli CFCS amongst all serovars. Specifically, *Salm*. Gallinarum 9 displayed the greatest susceptibility to *Lact. crispatus*, *Lact. reuteri* and *Lact. johnsonii*, with average inhibition zones of 23.66 ± 1.33 mm, 21.66 ± 2.53 mm, and 19.00 ± 1.63 mm, respectively. *Salm*. Gallinarum 287/91 showed the highest susceptibility to *Lact. crispatus*, *Lact. reuteri* and *Lact. johnsonii*, with average inhibition zones of 23.88 ± 3.24 mm, 23.22 ± 3.08 mm, and 21.55 ± 3.43 mm, respectively.

**Table 2:** Anti-*Salmonella* activity of lactobacilli CFCS as assessed by agar diffusion assay

Salmonella serovars	Zone of inhibition (mm)				
	MRS	Gentamicin	Lact. johnsonii	Lact. reuteri	Lact. crispatus
*Salm.* Typhimurium 4/74	0	12.88 ± 0.87	15.77 ± 0.62	15.00 ± 1.15	16.11 ± 1.83
*Salm*. Enteritidis P125109	0	13.66 ± 0.94	15.66 ± 0.47	15.44 ± 0.49	15.77 ± 1.22
*Salm*. Enteritidis AviPro^®^	0	14.55 ± 0.83	14.77 ± 1.03	14.44 ± 1.83	16.55 ± 1.83
*Salm*. Gallinarum 9	0	15.66 ± 2.82	19.00 ± 1.63	21.66 ± 2.53	23.66 ± 1.33
*Salm*. Gallinarum 287/91	0	17.44 ± 1.34	21.55 ± 3.43	23.88 ± 3.24	23.22 ± 3.08

Footnote: Data expressed as mean ± SD of triplicate experiments. Using MRS broth alone, as negative control group, there was zero (0) observed zone of inhibition. Gentamicin antibiotic discs (10 µg) were used as a zone of inhibition positive control

### Time-dependent Killing of Salmonellae Following Co-culture with Lactobacilli CFCS

**Fig. 1:**
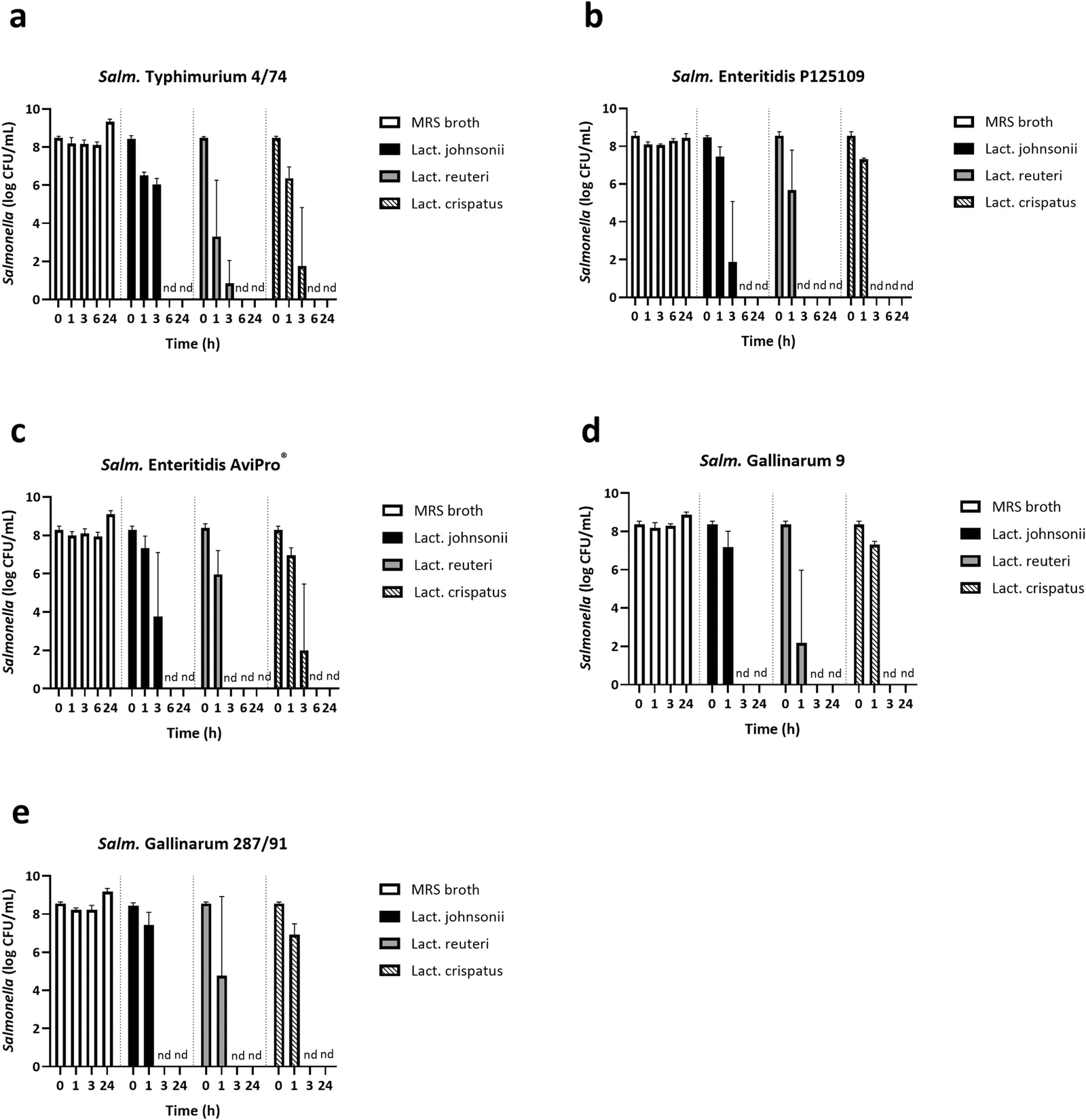
Time-dependent action of lactobacilli CFCS on viability of *Salmonella* serovars. One mL of each lyophilised, reconstituted (10X) lactobacilli CFCS (*Lact. johnsonii*, *Lact. reuteri* or *Lact. crispatus*) was combined with 1 mL of *Salmonella* culture (10^8^ CFU/mL) in LB broth; (**a**) *Salm*. Typhimurium 4/74, (**b**) *Salm*. Enteritidis P125109, (**c**) *Salm*. Enteritidis AviPro^®^, (**d**) *Salm*. Gallinarum 9, (**e**) *Salm*. Gallinarum 287/91. Samples (*N* = 3) were collected at 0, 1, 3, 6 and 24 h, and the number of *Salmonella* (log CFU/mL) was quantified in triplicate using the Miles and Misra method. nd, not detected

To further evaluate the antimicrobial activity of CFCS from *Lact. johnsonii*, *Lact. reuteri* and *Lact. crispatus* against *Salmonella* serovars, dynamic co-culture assays were conducted for up to 24 h in broth culture (Fig. 1). *Lact. reuteri* exhibited the highest inhibitory activity against *Salmonella*, compared to the effects of *Lact. crispatus* and *Lact. johnsonii*. Regarding variation among serovars, *Salm.* Gallinarum 9 and *Salm.* Gallinarum 287/91 showed greater susceptibility to lactobacilli compared to *Salm.* Typhimurium 4/74, *Salm.* Enteritidis P125109, and *Salm.* Enteritidis AviPro^®^. The results further reveal that all CFCS from lactobacilli completely inhibited *Salm.* Gallinarum 9 and *Salm.* Gallinarum 287/91 within 3 h following co-incubation in culture, while complete inhibition of *Salm.* Typhimurium 4/74, *Salm.* Enteritidis P125109, and *Salm.* Enteritidis AviPro^®^ occurred after 6 h post-incubation. Extending co-cultures to 24 h highlighted that all *Salmonella* serovars were completely inhibited. However, notable variations in inhibition were observed between strains. Specifically, *Salm.* Typhimurium 4/74 exhibited high susceptibility to *Lact. reuteri* CFCS, resulting in a reduction in viability from log 8 CFU/mL to log 1 CFU/mL within 3 h post-incubation. Concurrently, *Lact. crispatus* CFCS reduced the *Salmonella* cell counts to below log 2 CFU/mL, while co-culture with *Lact. johnsonii* CFCS resulted in approximately log 6 CFU/mL. Notably, *Lact. reuteri* CFCS showed exceptional antimicrobial effectiveness against both serovars of *Salm.* Gallinarum, reducing the count to below log 2 CFU/mL in *Salm.* Gallinarum 9 and log 4 CFU/mL in *Salm.* Gallinarum 287/91 within 1 h post-incubation. Additionally, CFCS from *Lact. crispatus* and *Lact. johnsonii* also exhibited robust antimicrobial activity, resulting in the complete termination of the *Salm.* Gallinarum serovars within 3 h post-incubation.

### Lactobacilli CFCS Reduce *Salmonella* Invasion into Chick Caecal Epithelial Cells

**Fig. 2:**
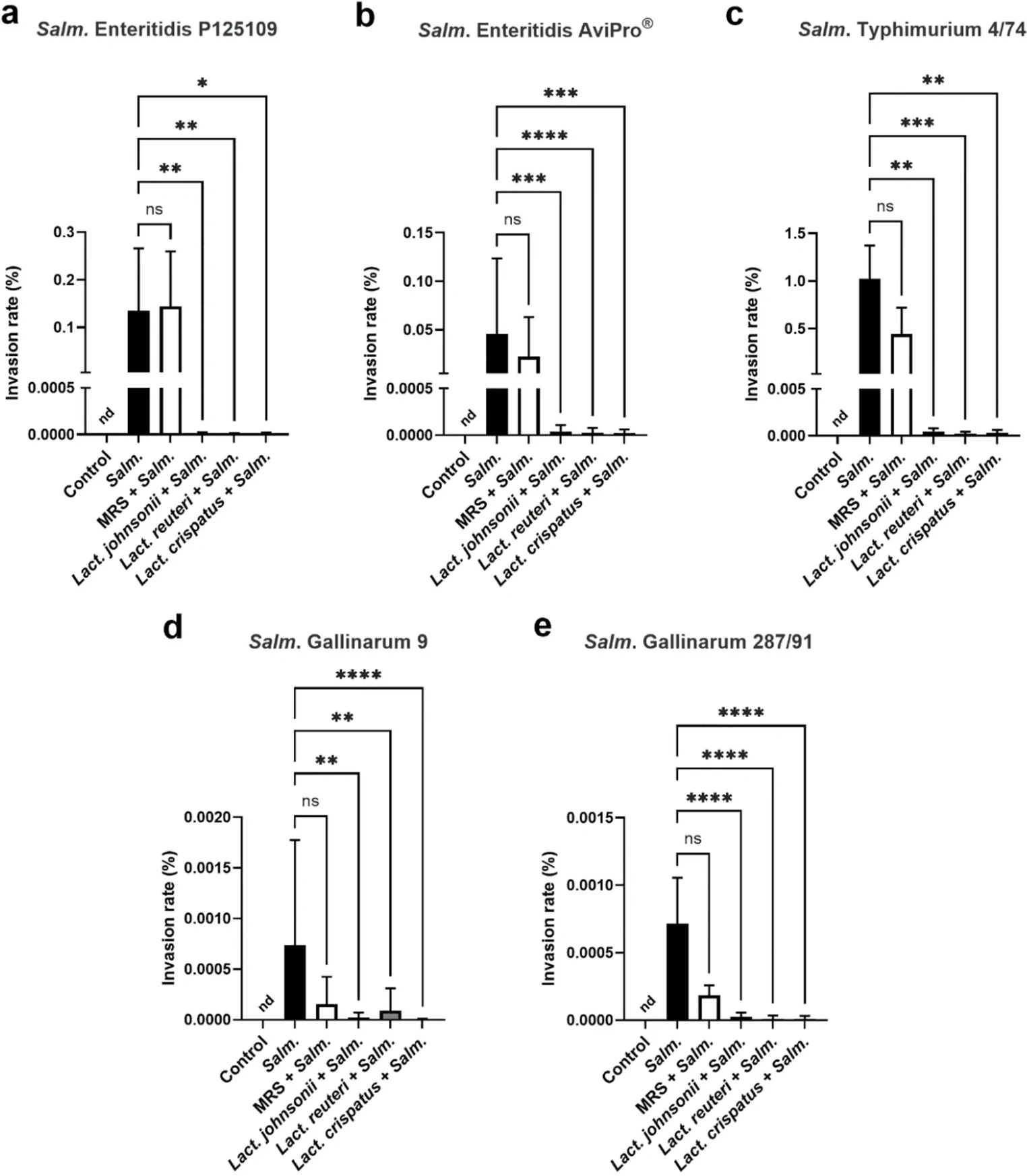
Invasion of *Salmonella* into chicken 8E11 caecal epithelial cells was diminished with CFCS from lactobacilli. *Salmonella* invasion rates (expressed as a % of the original inoculum of 10^6^ bacteria) were compared between (**a**) *Salm*. Enteritidis P125109, (**b**) *Salm*. Enteritidis AviPro®, (**c**) *Salm*. Typhimurium 4/74, (**d**) *Salm*. Gallinarum 9 and (**e**) *Salm*. Gallinarum 287/91 infected cells pre-treated for 2 h with 10X MRS broth, 10X CFCS from *Lact*. *johnsonii*, *Lact*. reuteri or *Lact*. *crispatus* (white bars), or without pre-treatment (black bars). Non-infected controls with culture medium alone. Differences between treatment groups were considered significant when *p < 0.05, **p < 0.01 and ***p < 0.001, using Kruskal-Wallis test followed by pairwise comparisons with Dunn’s post-hoc tests for multiple comparisons (N = 4, with triplicate counts per well). nd, not detected; ns, non significant difference

To identify the inhibitory effect of lactobacilli CFCS on *Salmonella* infection of chicken caecal 8E11 epithelial cell monolayers, the invasion rate after pre-treatment with each CFCS was evaluated compared to untreated, infected cells (Fig. 2). The results obtained showed that *Salmonella* serovars vary in their ability to invade the chicken 8E11 epithelial cell-line, with both *Salm*. Gallinarum serovars showing approximately 1,000 times lower invasion than the two *Salm*. Enteritidis serovars and *Salm*. Typhimurium 4/74. In addition, pre-treatment with lactobacilli CFCS significantly reduces the ability of *Salmonella* to invade chick 8E11 cells compared to the untreated group. The observed reduction in the cell invasion rate was clear across all groups pre-treated with lactobacilli CFCS. Compared to the untreated group, cells pre-treated with CFCS of *Lact. reuteri*, *Lact. crispatus*, and *Lact. johnsonii* exhibited a significant reduction in *Salm*. Typhimurium 4/74 invasion, from 1.02 (% of the original inoculum) in the untreated, infected control condition to 2.1 × 10^− 4^, 3.1 × 10^− 4^ and 4.4 × 10^− 4^% respectively (*N* = 4 replicates, with at least triplicate counts per well).

Similarly, both *Salm*. Enteritidis P125109 and *Salm*. Enteritidis AviPro^®^ showed a reduction in invasion rate compared to the untreated group. For *Salm*. Enteritidis P125109, the invasion rate in the untreated condition was 0.13 × 10^− 2^% of the original inoculum. In comparison, the invasion rates with pre-treatment using *Lact. reuteri*, *Lact. crispatus*, and *Lact. johnsonii* were 6.17 × 10^− 6^, 8.28 × 10^− 6^ and 9.34 × 10^− 6^%, respectively. For *Salm*. Enteritidis AviPro^®^, the invasion rate was reduced from 4.57 × 10^− 2^% in the untreated condition to 2.23 × 10^− 5^, 2.39 × 10^− 5^ and 3.97 × 10^− 5^% with pre-treatment using *Lact. crispatus*, *Lact. reuteri* and *Lact. johnsonii*, respectively. Regarding *Salm*. Gallinarum 9, the invasion rate of this serovar was observed to decrease from 7.37 × 10^− 4^ to 2.87 × 10^− 6^, 8.86 × 10^− 5^ and 2.34 × 10^− 5^ with pre-treatment by *Lact. crispatus*, *Lact. reuteri*, and *Lact. johnsonii*, respectively. Pre-treatment with lactobacilli CFCS also significantly reduced the infection rate of *Salm.* Gallinarum 287/91, from 7.14 × 10^− 4^ in the untreated group to 8.85 × 10^− 6^, 1.04 × 10^− 5^ and 2.04 × 10^− 5^ with pre-treatment by *Lact. reuteri*, *Lact. crispatus* and *Lact. johnsonii*, respectively.

Results from the LDH cytotoxicity assay demonstrated that all lactobacilli CFCS-treated groups exhibited low or negligible cytotoxicity, with some displaying cytoprotective effects (Supplementary Information file S2). Compared to the baseline LDH levels in chicken 8E11 intestinal epithelial cells, treatment with 10X CFCS from *Lact. johnsonii* and 10X MRS broth resulted in minimal cytotoxicity. In contrast, treatment with 10X CFCS from *Lact. crispatus* and *Lact. reuteri* exhibited cytoprotective effects, reducing LDH release by 7.62 ± 1.75% and 12.44 ± 1.8%, respectively (*N* = 4, with triplicate measurements made within the assay).

### Lactobacilli CFCS Ameliorates *Salmonella*-induced TNF Release from Macrophages

**Fig. 3:**
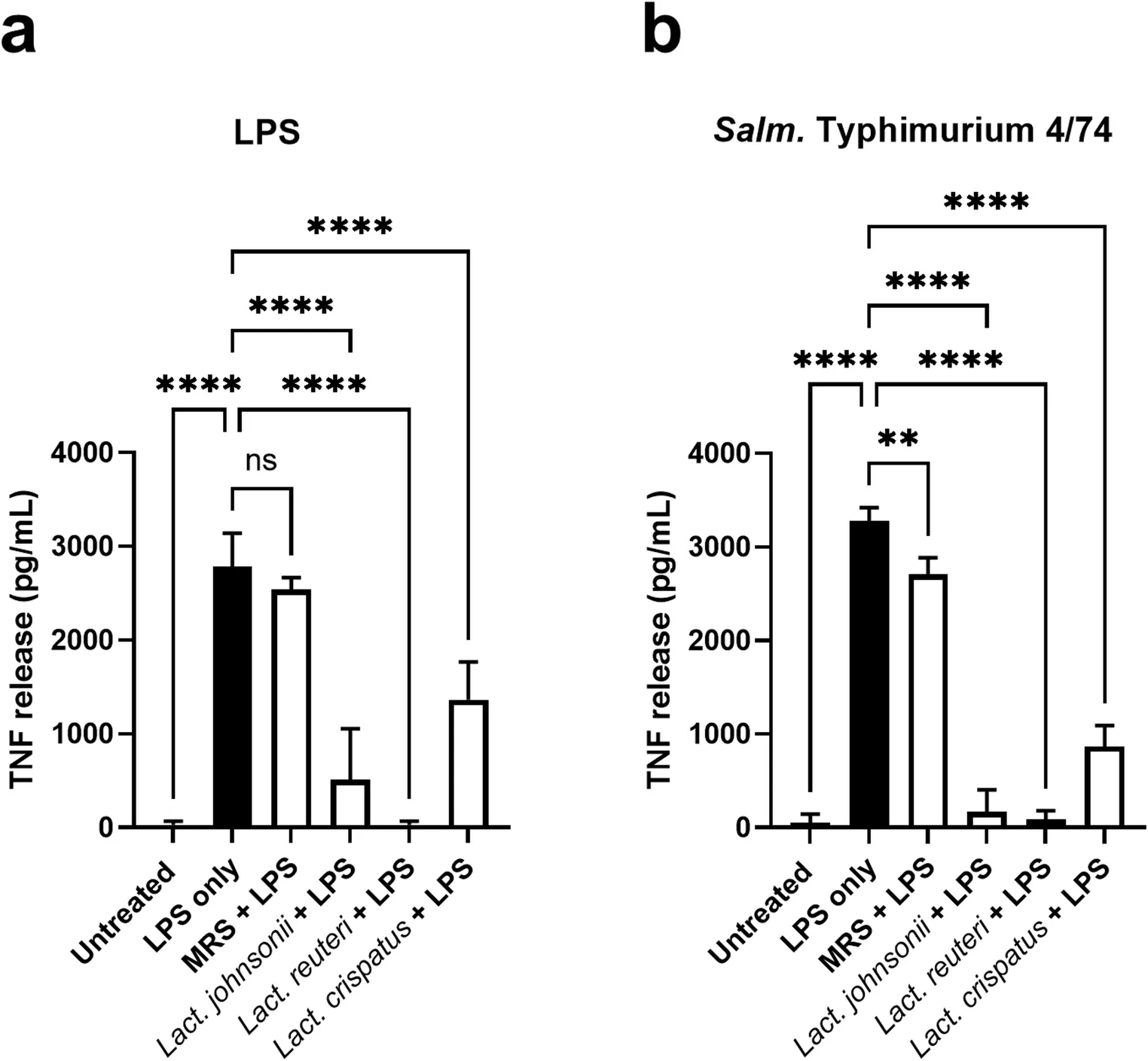
Action of lactobacilli CFCS on *Salmonella*-induced pro-inflammatory TNF release from murine macrophages. TNF levels released to medium (measured by ELISA) of murine J774.A1 macrophages challenged with (**a**) *Salm.* Typhimurium lipopolysaccharide (LPS; 50 ng/mL, for 1 h) or (**b**) *Salm*. Typhimurium 4/74 (MOI 10, for 2 h) with or without pre-treatment with lactobacilli 10X CFCS or 10X MRS broth. Statistical analysis was conducted using One-way ANOVA, followed Tukey’s multiple comparisons tests. Significant differences, **p < 0.01 and ****p < 0.0001 (*N* = 4); ns, non significant difference

Pre-treatment with lactobacilli 10X CFCS prior to 50 ng/mL *Salmonella* LPS treatment or exposure to *Salmonella* Typhimurium 4/74 infection (MOI 10) ameliorated release of pro-inflammatory cytokine TNF from macrophages. TNF levels released in response to LPS were observed to be 2783 ± 360.3 pg/mL compared to unstimulated controls (28.5 ± 41.1 pg/mL; *p* < 0.0001; One way ANOVA, *N* = 4). LPS-induced TNF levels were diminished in all the lactobacilli 10X CFCS pre-treatment groups, with *Lact. reuteri* showing reductions of 99%, *L. johnsonii* 81.6% and *Lact. crispatus* 50.9%, (all *p* < 0.0001) with no significant reduction seen with 10X MRS broth; see Fig. 3a. In response to *Salmonella* infection, the TNF levels released from macrophages were observed to be 3281.6 ± 139.6 (mean ± SD) pg/mL compared to uninfected controls at 54.1 ± 92.1 pg/mL (*p* < 0.0001; One way ANOVA, *N* = 4). Similarly, pre-treatment with 10X CFCS from *Lact. reuteri* lowered TNF release by 97.3%, with *Lact. johnsonii* and *Lact. crispatus* reducing TNF release by 94.8% and 73.5%, respectively (all *p* < 0.001); see Fig. 3b. A small, but significant, reduction (17.5%) in TNF release induced by *Salmonella* was also observed using 10X MRS broth alone (*p* < 0.01).

Characterisation of the anti-microbial activity of *Lactobacillus* CFCS reveals low molecular weight moieties likely of an acidic nature, intolerant to heat and resistant to proteases

#### Antimicrobial Compounds in Lactobacilli CFCS are Low Molecular Weight Moieties

Molecular characterisation of CFCS from the three selected lactobacilli strains using Amicon^®^ size-exclusion centrifugal ultrafiltration, with filtrates and retained components subjected to agar diffusion assays using serovar *S.* Typhimurium 4/74, suggest that the anti-*Salmonella* bioactive compounds produced by lactobacilli are primarily low-molecular weight metabolites, ≤ 3 kDa (Supplementary Information file S3). The ultracentrifugation filtrates of the CFCS of *L. johnsonii* and *L. crispatus* obtained from the 30 kDa and 10 kDa cartridges showed full antimicrobial activity, comparable to unfractionated CFCS. For CFCS of *L. reuteri*, some anti-*Salmonella* activity was found in the retained material obtained from the 10 kDa filter cartridge. Using a 3 kDa cutoff filter, the antimicrobial activity of all three lactobacilli CFCS was primarily detected in the pass-through fraction, with some residual activity remaining in the retained fraction. This suggests that the antimicrobial compounds are predominantly ≤ 3 kDa. Sephadex G25 gel filtration chromatography confirmed that these metabolites are small molecular weight molecules (Supplementary Information file S4).

#### Antimicrobial Metabolite Activity within Lactobacilli CFCS is pH Dependant

The average pH of overnight microaerobic cultures of lactobacilli CFCS (1X) ranged between 3.98 and 4.4 (*N* = 6 independent lactobacilli cultures), whereas MRS broth alone had a pH of 5.23 ± 0.03 (mean ± SD). The average pH of lyophilized (10X) lactobacilli CFCS was 4.84 ± 0.03 for *Lact. johnsonii*, 4.19 ± 0.02 for *Lact. reuteri* and 4.31 ± 0.06 for *Lact. crispatus* (*N* = 3 independent lactobacilli cultures). The pH of lyophilized MRS broth was 5.91 ± 0.13 (*N* = 3).

**Fig. 4:**
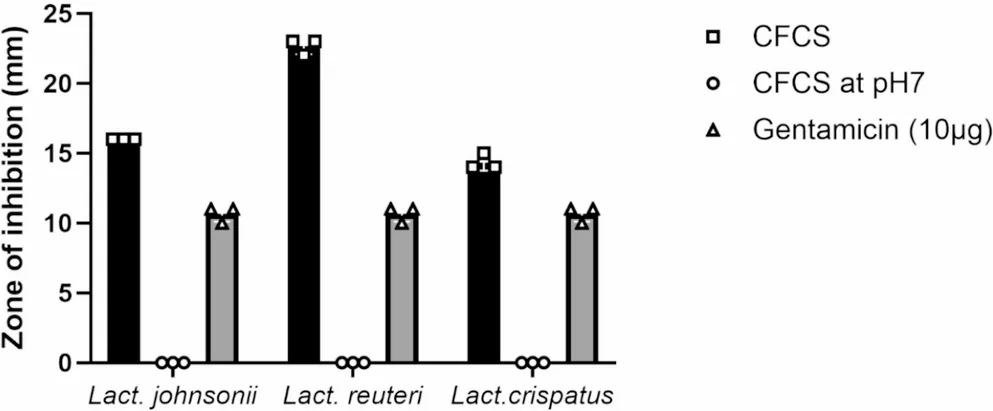
Loss of anti-*Salmonella*activity of lactobacilli CFCS neutralised to pH 7. Lactobacilli CFCS was adjusted to pH 7, lyophilised and reconstituted (10X) and tested for anti-*Salm.* Typhimurium 4/74 activity by agar diffusion assay. Data reported as mean ± SD (*N* = 3). Standard (pH unadjusted) 10X CFCS from each *Lactobacillus* spp. overnight microaerobic culture, and 10 µg Gentamicin antibiotic discs were used as inhibition positive controls

Neutralising the mildly acidic pH of each lyophilised lactobacilli CFCS (i.e. adjusting to pH 7) resulted in complete loss of their anti-*Salmonella* activity (Fig. 4). In contrast, 10X CFCS of *Lact. reuteri*, *Lact. johnsonii* and *Lact. crispatus* at their normal pH exhibited inhibition zones in the agar well diffusion assay of 22 mm, 16 mm and 14 mm, respectively.

#### Postbiotic Antimicrobial Activity of lactobacilli CFCS is Resistant to Heat Treatment

**Fig. 5:**
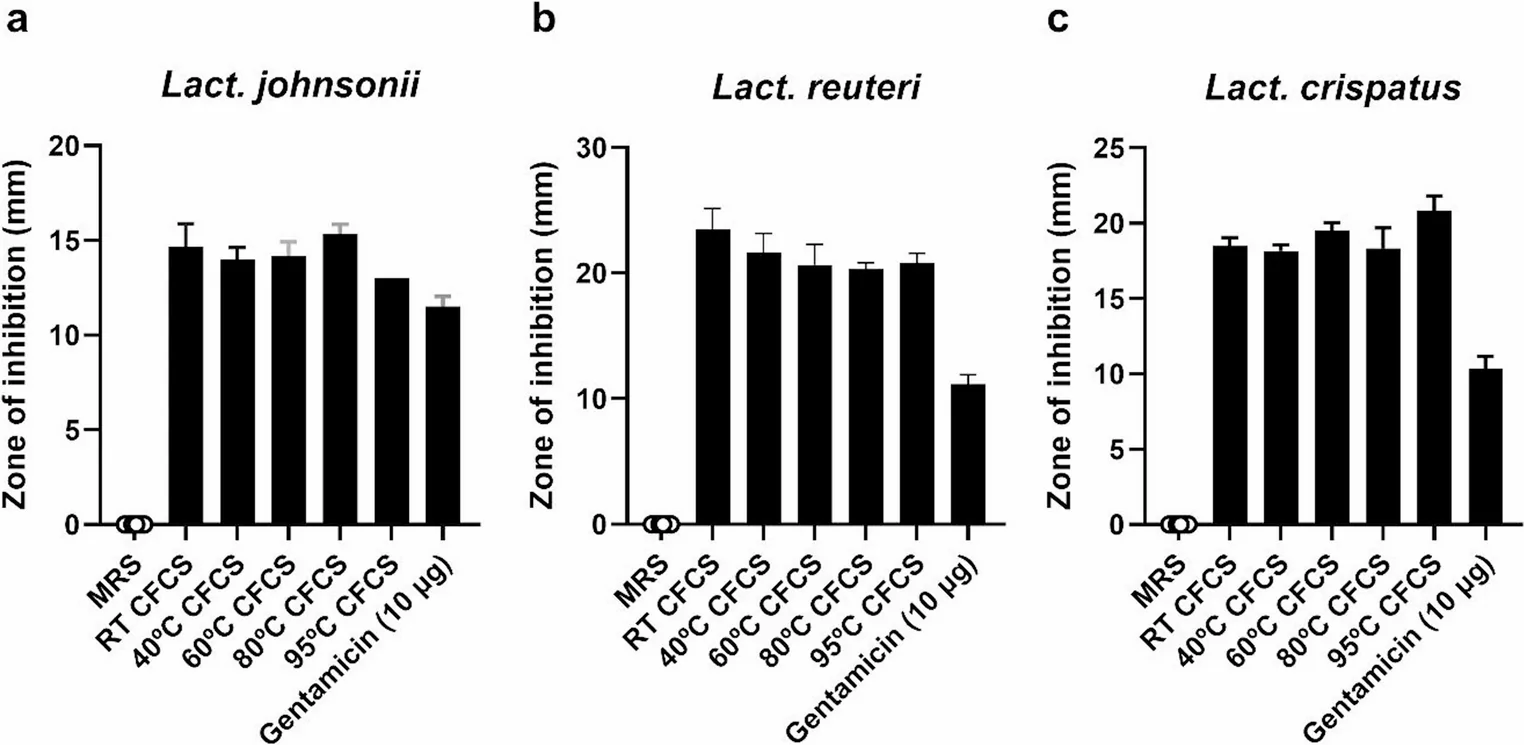
Antimicrobial activity of lactobacilli CFCS is stable following heat treatment. Lactobacilli were cultured overnight under microaerobic conditions, lyophilised and reconstituted in 1 mL sterile deionised water (10X CFCS). Samples of 10X CFCS from (**A**) *Lact. johnsonii*. (**B**) *Lact. reuteri*. (**C**) *Lact. crispatus* were subjected to heat treatment at 40 °C, 60 °C, 80 °C and 95 °C, for 30 min, compared to 10X CFCS at room temperature (RT). Samples were tested against *Salmonella* Typhimurium 4/74 in agar diffusion assays, with 10 µg Gentamicin discs used as inhibition positive controls, and 10X MRS broth alone (*N* = 6)

The observed tolerance of antimicrobial components/metabolites within lactobacilli CFCS to different levels of heat treatment can be seen in Fig. 5. The results indicated that antimicrobial activity of all three lactobacilli CFCS against *S.* Typhimurium 4/74 was not eliminated by the heat, with postbiotic antimicrobial activity showing bioactive stability throughout a range of temperatures tested, from 40 to 95 °C, compared to CFCS at room temperature (see Fig. 4.2). Even extreme high pressure, steam heat treatment (autoclave, at 121 °C) could not diminish postbiotic anti-*Salmonella* activity of lactobacilli CFCS (see Supplementary Information S5).

#### Antimicrobial activity of lactobacilli CFCS does not appear to be proteinaceous in origin

The antimicrobial activity of lactobacilli 10X CFCS was not affected by the action of proteolytic enzymes. Both pepsin- and papain-treated lactobacilli CFCS showed no significant differences in activity against *Salm.* Typhimurium 4/74 when compared to that observed with untreated lactobacilli CFCS; *N* = 3 (see Supplementary Information file S6). To further assess for any proteinaceous contribution to postbiotic anti-*Salmonella* activity in the lactobacilli CFCS, a common method of purification and concentration of bacteriocins/small anti-microbial peptides was conducted using ethyl acetate solvent-based extraction. The extraction process resulted in the separation of *Lact. johnsonii* and *Lact. reuteri* CFCS into three fractions; an upper solvent ‘extraction’ phase, an emulsion phase and a lower ‘retained’ aqueous phases, whilst the CFCS of *Lact. crispatus* was divided only into upper and lower phase fractions, with no emulsion layer evident. The ethyl acetate ‘extraction’ fractions of all three lactobacilli CFCS exhibited no anti-*Salmonella* activity. The lower ‘retained’ aqueous fraction of *Lact. reuteri* exhibited the highest anti-*Salmonella* activity, with an inhibition zone of 20.33 ± 0.47 mm, followed by *Lact. johnsonii* at 20 mm and *Lact. crispatus* at 12.33 ± 0.94 mm (*N* = 3). Additionally, the emulsion fractions obtained from the extraction of CFCS from *Lact. johnsonii* and *Lact. reuteri* both showed some antimicrobial activity, each with an inhibition zone of 16 ± 1.63 mm. Solvent extracted MRS broth alone, showed no inhibitory activity in the upper, emulsion and lower phase fractions tested (see Supplementary Information file S6). Overall, no bacteriocins/small AMPs appear to be present or bioactive in lactobacilli CFCS.

### HS-SPME-GC/MS analysis identifies key volatile organic compounds (VOCs) in probiotic lactobacilli CFCS

**Fig. 6:**
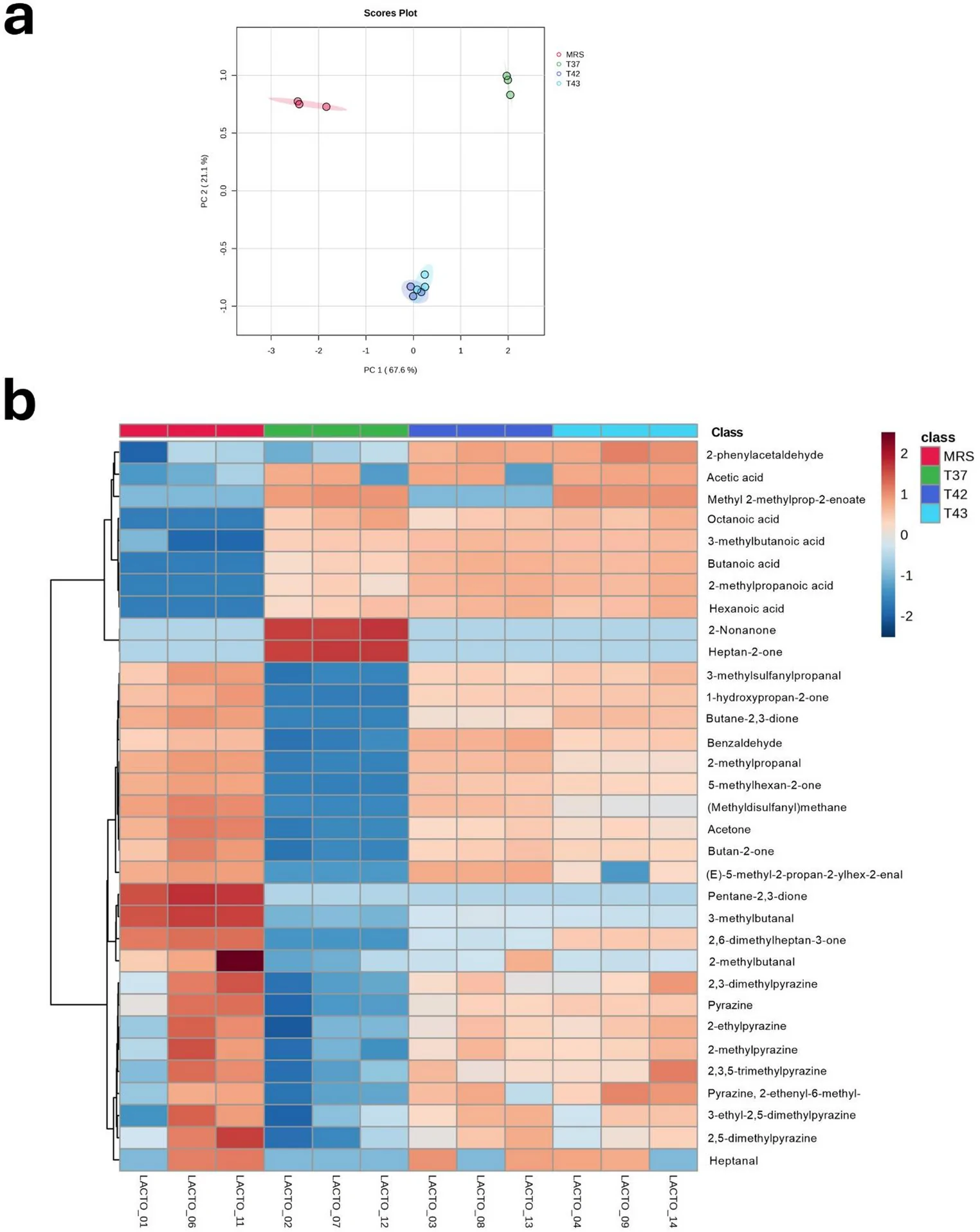
Volatile organic compound (VOC) metabolome of lactobacilli CFCS. (**a**) Principal component analysis (PCA) score plot of the VOC metabolome from CFCS of overnight cultures of lactobacilli *Lact. johnsonii* (T37), *Lact. reuteri* (T42) and *Lact. crispatus* (T43), compared to MRS broth alone (*N* = 3 independent cultures for each group). (**b**) Heatmap illustrating hierarchical clustering analysis of differentially abundant VOCs observed in CFCS of overnight cultures from lactobacilli identified by HS-SPME-GC/MS analysis. The heatmap colour scale illustrates the relative concentrations of the differential VOC identified. Dark red indicates a relatively high concentration of the compound, with dark blue indicating a relatively low concentration

To identify the VOC metabolome produced in CFCS from overnight culture of lactobacilli *Lact. johnsonii*, *Lact. reuteri* and *Lact. crispatus*, multivariate data analysis was conducted. Principal Component Analysis (PCA) revealing that the CFCS of all lactobacilli groups were clearly separated from the MRS broth control, with *L. reuteri* and *L. crispatus* positioned close to each other, while *Lact. johnsonii* stood apart (Fig. 6a**)**. The PCA accounted for 88.7% of the variance, with PC1 contributing 67.6% and PC2 contributing 21.1%. Loading scores for PC1 and PC2 are available in Supplementary Information file S7. The differential abundance of metabolic profiles between lactobacilli CFCS and the culture medium is illustrated in the heat map (Fig. 6b). A total of 33 VOCs were identified in the CFCS from all three lactobacilli. Of these, 28 compounds were established as being significantly different between groups (*p* < 0.05; one-way ANOVA, followed by Tukey’s HSD post-hoc tests); see Supplementary Information file S7).

**Fig. 7:**
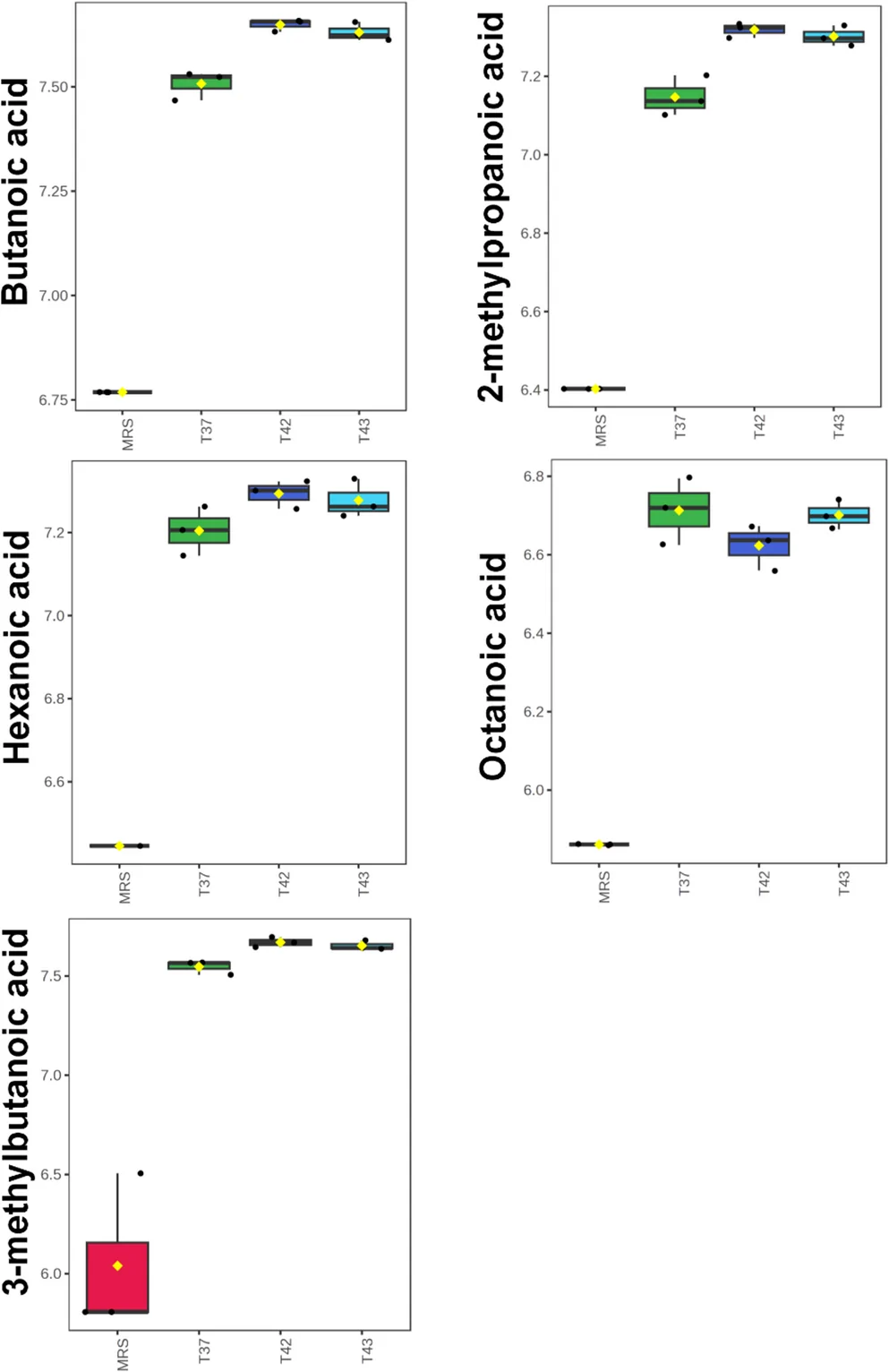
Comparison of organic acids levels identified from the VOC metabolome of lactobacilli. Normalised abundance of organic acids identified by HS-SPME-GC/MS in 10X CFCS of *Lact. johnsonii* (T37), *Lact. reuteri* (T42) and *Lact. crispatus* (T43), compared to MRS broth alone (*N* = 3 independent cultures for each group). *Significant differences in normalised organic acid abundance at *p* < 0.05 compared to MRS broth, as determined by one-way ANOVA, followed Tukey’s HSD post-hoc testing for multiple comparisons

Compounds identified were 9 ketones, 7 aldehydes, 6 pyrazines, 1 aldehyde and 5 organic acids. The latter group included SCFAs and medium-chain fatty acids (MCFAs), such as butanoic acid, 2-methylpropanoic acid, 3-methylbutanoic acid, hexanoic acid and octanoic acid (see Fig. 7). The VOC metabolome of the Sephadex G25 gel column chromatography fractions of lactobacilli CFCS harbouring anti-*Salmonella* activity identified a total of 35 metabolites, with 23 found to be significantly different between groups (*p* < 0.05). The differential metabolites included 6 ketones, 2 aldehydes, 2 alcohols, 7 pyrazines and 6 organic acids (including additionally acetic acid); see Supplementary Information file S8.

## Discussion

Poultry and egg products are major sources of *Salmonella* infections in humans. Various efforts have been made to control pathogen transmission in poultry production including use of lactic acid-producing bacteria (LAB) as gastrointestinal health enhancers, promoting immunity and control of enteric pathogens. However, understanding the functional application of probiotics remains limited due to strain specific variations in viability, ability to colonize the host and in antimicrobial activity, which greatly impact their probiotic properties.

Having identified three lactobacilli from caecal content of mature, healthy broiler chickens —*Lact. johnsonii* (T37), *Lact. reuteri* (T42) and *Lact. crispatus* (T43)— in vitro screening for anti-*Salmonella* activity was performed on the CFCS produced from these strains. MIC, MBC and assessment of anti-*Salmonella* activity by co-culture and agar well diffusion assay approaches showed that the CFCS from all three lactobacilli exhibited promising antibacterial activity against five key serovars — *Salm.* Typhimurium 4/74, *Salm*. Enteritidis P125109, *Salm*. Enteritidis AviPro^®^, *Salm*. Gallinarum 9 and *Salm*. Gallinarum 287/91. MIC and co-culture assays revealed *Lact. reuteri* CFCS exhibiting the highest anti-bacterial activity, followed by *Lact. crispatus* and *Lact. johnsonii*. *Salm*. Gallinarum showed the highest susceptibility to all three lactobacilli CFCS, followed by *Salm.* Enteritidis and *Salm.* Typhimurium. MIC ranged from 8.98 to 18.58 mg/mL, and MBC from 12.62 to 25.54 mg/mL across all the lactobacilli CFCS. Given the MBC/MIC ratio was ≤ 4, the activity would be considered bactericidal, rather than bacteriostatic [31]. Supporting the MIC data, the CFCS co-culture assays confirmed *Salm.* Gallinarum as being completely inhibited by Supporting the MIC data, the CFCS co-culture assays confirmed *Salm.* Gallinarum as being completely inhibited by lactobacilli CFCS within 3 h of incubation, whereas *S*. Enteritidis was inhibited between 3 and 6 h, depending on the lactobacilli under test, while *Salm*. Typhimurium 4/74 was inhibited within 6 h by all CFCS irrespective of the lactobacilli cultured. All serovars were inhibited after 24 h co-culture highlighting sustained anti-microbial activity of compounds within the CFCS. Results from the agar well diffusion assays supported that *Salm.* Gallinarum serovars were more susceptible to CFCS from lactobacilli, than other serovars. Previous studies have reported diverse inhibitory effects of *Lactobacillus* postbiotics against *Salmonella* serovars Typhimurium, Typhi, Enteritidis, Heidelberg and Gallinarum [11, 40, 55]. Our study also underscores the diverse susceptibility of *Salmonella* to lactobacilli CFCS, alongside the varying antimicrobial activity potential observed amongst different lactobacilli.

Intestinal epithelial cell invasion is a critical process for the initiation of *Salmonella* pathogenesis, and inhibiting this step offers a promising way to prevent infection. There was variation seen in the ability of *Salmonella* serovars to invade 8E11 chick enterocytes, likely influenced by the diverse expression of key virulence factors seen among *Salmonella* strains [17, 59]. Importantly, we observed significant reduction in *Salmonella* invasion of 8E11 cell monolayers following treatment with CFCS from all three lactobacilli, highlighting their potential as probiotics. This is in concordance with a previous study demonstrating CFCS of *Lact. acidophilus* inhibition of cell invasion and motility impairment of *Salm.* Typhimurium [51]. Moreover, we noted that even high concentrations of CFCS from all three lactobacilli did not result in 8E11 enterocyte cytotoxicity, with CFCS from *Lact. reuteri* and *Lact. crispatus* exhibiting cytoprotection. Conversely, there is evidence from other studies that show that high concentrations of CFCS from other lactobacilli (e.g. *Lactiplantibacillus plantarum*) may exhibit cytotoxic effects on various cell types, including cancer cell-lines [10, 63]. Such variation observed amongst different strains of lactobacilli should be carefully considered for specific probiotic applications to mitigate any potential cytotoxic effects of concentrated postbiotic preparations.

TNF is an essential response cytokine against infection, helping to activate/coordinate mucosal immune responses immune cells (macrophages, neutrophils and T cells) and promote bacterial clearance, but high levels can also contribute to the inflammation and tissue pathology seen in salmonellosis [4]. We showed that CFCS from lactobacilli significantly reduced LPS- or whole bacteria-induced TNF release from murine macrophages, with CFCS from *Lact. reuteri* showed the highest activity, followed by *Lact. johnsonii* and *Lact. crispatus*. A small but statistically significant reduction (17.5%) in *Salmonella*-induced TNF release was also observed with 10X MRS broth alone, suggesting that certain components present in the concentrated culture medium may exert a degree of immunomodulatory activity. Specific amino acids present in MRS broth, such as glutamine and arginine, have been reported to suppress TNF production in immune cells [64, 66]. However, the magnitude of TNF reduction observed with 10X MRS broth alone was substantially lower than that observed with 10X CFCS from lactobacilli, indicating that the anti-inflammatory effect of CFCS is primarily attributable to bacteria-derived metabolites rather than components of the broth itself. Other recent studies have highlighted that postbiotics derived from lactobacilli, such as *Lacticaseibacillus acidophilus* and *Lacticaseibacillus casei*, can exhibit anti-inflammatory properties by reducing the secretion of TNF, whilst also promoting the production of anti-inflammatory cytokines such as interleukin 10 (IL-10) [53, 86]. This underscores lactobacilli postbiotics as promising agents in balancing antimicrobial defence with inflammation control, to benefit gut health.

Molecular characterisation of the CFCS revealed that the antimicrobial activity of metabolites produced by the three lactobacilli was abolished when neutralised to pH 7, indicating that these postbiotics function optimally under mildly acidic conditions, pH range 3.9–4.9. The active antimicrobial compounds of these lactobacilli are likely not limited to lactic acid, with several studies reporting strain-specific non-lactic acid antimicrobial molecules [15, 72]. The results from our heat tolerance studies undertaken here on the lactobacilli CFCS, demonstrated conclusively that the antimicrobial metabolites generated are heat stable, similar to studies on CFCS from *Lacticaseibacillus paracasei*, *Lacticaseibacillus casei* and *Lactiplantibacillus brevis* [30, 36, 37]. Several studies have reported heat-resistant anti-microbial proteins generated by lactobacilli and that antimicrobial activity could be abolished after enzymatic treatment with papain, pepsin, proteinase K or trypsin [47, 68]. Proteolytic digestion experiments here confirmed the antimicrobial activity of the CFCS from *Lact. johnsonii*, *Lact. reuteri* and *Lact. crispatus* was not attributable to heat resistant protein/peptides, and ethyl acetate extraction analysis, utilised to concentrate any small proteins/peptides, also confirmed no such moieties with anti-*Salmonella* activity were present, or bioactive, in the CFCS of all three lactobacilli.

Molecular cut-off centrifugal ultrafiltration and gel chromatography further revealed that the antimicrobial metabolites from all three lactobacilli were molecules ≤ 3 kDa in size. Considering this, HS-SPME-GC-MS analysis successfully identified several candidate antimicrobial VOCs in the CFCS, including organic acids, aldehydes, ketones and pyrazines. The VOC profile of *Lact. reuteri* and *Lact. crispatus* appeared closely related, despite utilising different fermentation pathways; obligate heterofermentative (generating lactic acid along with other metabolites such as ethanol, carbon dioxide and acetic acid) versus obligate homofermentative (producing primarily lactic acid), respectively. In contrast, *Lact. johnsonii*, which shares the same fermentation pathway as *Lact. crispatus*, stood out as distinct, suggesting that metabolite production in lactobacilli is unique and cannot be solely predicted by their fermentation pathways. SCFAs and MCFAs were the primary organic acids found in the lactobacilli CFCS, including acetic acid, butanoic acid, 2-methylbutanoic acid, 3-methylbutanoic acid, hexanoic acid and octanoic acid. Of note, higher levels of hexanoic and octanoic acids were identified in the VOC metabolome produced by *Lact. reuteri*, which may, in part, account for its superior inhibitory effect observed in our bioassays. These organic acids are well documented as having bactericidal activity by reducing environmental pH and inducing intracellular accumulation of anions [8, 28, 79]. Organic acid chain length, side chains, hydrophobicity and pKa values impact on antimicrobial activity [81]. MCFAs, with their higher pKa values compared to SCFAs, are notably more effective against bacteria, including *Salmonella* [25, 80]. Hexanoic acid (caproic acid) has been reported as the most potent MCFA against *Salm.* Enteritidis, suppressing intestinal epithelial cell invasion, and decreasing colonization of the caeca and internal organs in chickens [79]. Several studies have indicated the effect of octanoic acid (caprylic acid) on *Salmonella* mitigation [13, 46]. Feed supplementation with octanoic acid can reduce *Salmonella* abundance in the intestine and internal organs of chicken, partly by inhibiting epithelial cell invasion through downregulation of *Salmonella* invasion-associated genes *hil*A and *hil*D [38]. However, the functional ability of lactobacilli to combat *Salmonella* is influenced by multiple factors, leading to variations in strain responses seen against *Salmonella* serovars [12]. Treatment with butanoic acid can reduce the expression of *Salmonella* pathogenicity island 1 (SPI-1), resulting in decreased bacterial virulence, shedding and caecal colonisation in chickens [20, 39, 81]. In addition, 2-methylbutanoic acid (2-methylbutyric acid) and 3-methylbutanoic acid (isovaleric acid), are noted for their antibacterial properties against *Salm.* Typhimurium LT-2 [26]. Studies have also reported the inhibitory effect of acetic acid against *Salmonella* [7, 76]. However, acetic acid is known to upregulate SPI-1, which increases the bacterial function in invading intestinal epithelial cells. Therefore, the concentration ratio between acetic acid and butanoic acid appears to have a significant impact on *Salmonella* infection and colonization [39].

Other key VOCs identified in the CFCS, included aldehydes, ketones and pyrazines. Aldehydes and their derivatives are known to have potent antimicrobial, antioxidative, anti-inflammatory and immunomodulatory properties [3]. In this study, 3-(methylsulfanyl)propanal and benzaldehyde were identified as significant compounds in the CFCS of our three lactobacilli. Benzaldehyde has been shown to exhibit significant antibacterial activity against *Salmonella* [18], with 3-(methylsulfanyl)propanal also reported to harbour anti-microbial activity [43]. *Lact. reuteri* are known to produce the antimicrobial aldehyde Reuterin (3-hydroxypropionaldehyde), but not all strains do so [75]. Reuterin synthesis though depends on the presence of specific genes and optimal environmental conditions for their expression, with glycerol identified as being particularly important [83, 90]. Here, we cultured all three lactobacilli in MRS broth without glycerol supplementation and thus these growth conditions may not have supported our *Lact. reuteri* strain in producing Reuterin. Also identified as major VOCs in the anti-*Salmonella* inhibitory fractions of lactobacilli CFCS were several ketones (including heptane-2,3-dione, 1-hydroxypropan-2-one, acetone, butane-2,3-dione, pentane-2,3-dione, and 2,6-dimethylheptane-3-one) and pyrazines (2,5-dimethylpyrazine, 2,3,5-trimethylpyrazine, 2-ethyl-6-methylpyrazine, 3-ethyl-2,5-dimethylpyrazine, 2-ethylpyrazine, pyrazine and 2-methylpyrazine). Although information is fairly limited in the literature, the current understanding suggests that ketones such as heptan-2-one, butan-2-one, butane-2,3-dione and acetone may function as antimicrobial agents by inhibiting bacterial drug efflux, promoting ROS production and damaging bacterial cell structures [21, 27]. Pyrazine and its derivatives, particularly 2,3-dimethylpyrazine and 2,5-dimethylpyrazine, also demonstrate significant broad-spectrum antibacterial activity [9]. Future research should help uncover the specific activities of these VOCs and any possible synergistic interactions contributing to the antimicrobial actions of lactobacilli CFCS.

## Conclusions

This study has demonstrated the potent anti-*Salmonella* activity of CFCS from three lactobacilli (*Lact. johnsonii*, *Lact. reuteri*, and *Lact. crispatus*) including bactericidal effects, inhibition of pathogen-host intestinal epithelial cell invasion, ability to modulate host immune inflammatory response to infection, and production of volatile organic anti-microbial metabolites. The inhibitory effects and the extent of their activity varied depending on both the *Lactobacillus* strain and the *Salmonella* serovar, reflecting a complex interplay between probiotic and pathogenic strains. The major volatile metabolite compounds identified within the *Lactobacillus* CFCS were organic acids, including MCFAs and SCFAs, aldehydes, alcohols, ketones and pyrazines. All *Lactobacillus* strains exhibit excellent potential as probiotics for controlling *Salmonella* in chickens. Overall, this study confirms that *Lactobacillus* strains isolated from the mature, healthy broiler caecal microbiota exhibit promising abilities to outcompete significant *Salmonella* serovars relevant to the poultry industry, including *Salm.* Typhimurium, *Salm.* Enteritidis, and *Salm*. Gallinarum. It will be important to examine whether these lactobacilli supplemented to poultry, either individually, or as a probiotic cocktail, can effectively inhibit salmonellosis, examining for reduction of *Salmonella* colonisation of lower intestinal tract and caecum, reduction in spread to the liver and spleen, and reduction of shedding.

## Supplementary Information

Below is the link to the electronic supplementary material.

Supplementary Material 1 (DOCX 22.8 KB)

Supplementary Material 2 (DOCX 84.9 KB)

Supplementary Material 3 (DOCX 383 KB)

Supplementary Material 4 (DOCX 596 KB)

Supplementary Material 5 (DOCX 215 KB)

Supplementary Material 6 (DOCX 457 KB)

Supplementary Material 7 (DOCX 29.3 KB)

Supplementary Material 8 (DOCX 301 KB)

## Data Availability

The original contributions presented in the study are included within the article and the Supplementary Information files. Further inquiries can be directed to the corresponding author.
