# Supplementary material for: Lactobacilli Isolated from the Caecum of Healthy Broilers with Antimicrobial Activity are Probiotic Candidates for Controlling Salmonella
Source: Probiotics Antimicrob Proteins. 2026 Mar 19;18(6):7723–43. doi: 10.1007/s12602-026-10980-5 (PMC13518464; doi:10.1007/s12602-026-10980-5)
Supplement: Supplementary file 1 [file 12602_2026_10980_MOESM1_ESM.docx]

**Supplementary Information file S1**

**Isolation and identification of bacteria species from healthy chicken caecal contents**

**Methods S1**:

Caecal content was collected from healthy 7-week-old Ross 308 Broilers sacrificed by cervical dislocation. Contents were pooled and suspended into sterile phosphate-buffered saline (PBS; 1:20 w/v) and the suspension was filtered through a coarse 25 µm Whatman filter (Sigma-Aldrich, UK) to produce microbiome transplant material, as described previously (Pottenger et al., 2023). Broiler chicks were hatched and housed in the University of Liverpool high biosecurity facility. All work was carried out in accordance with UK legislation governing use of experimental animals under project license number PPL 40/3652 and was reviewed and approved by the University of Liverpool (Liverpool, UK) ethical review process.

A lawn of the transplant preparation was spread onto nine Columbia Blood Agar (CBA) plates supplemented with 5% sheep blood. Three of each were incubated aerobically (37°C), microaerobically (42°C) and anaerobically (37°C) for up to 48 hours. In addition to CBA, the transplant preparation was also cultured on Reinforced Clostridial Agar (RCA) and De Man, Rogosa and Sharpe (MRS) agar under anaerobic conditions at 37 °C for up to 48 h. Plates were observed for morphologically distinct colony growth, with each differentially identified colony being sub-cultured onto their respective growth media and incubated for a further 48 h in their respective growth conditions to obtain pure, well isolated colonies which were stored in Microbank bead vials (ProLab Diagnostics; Bromborough, UK).

Isolate identification was performed using MALDI-TOF mass spectrometry (Veterinary Microbiology Diagnostic Laboratory, University of Liverpool, Leahurst Campus, Neston, UK). Whole genome sequencing was also carried out for selected isolates by the MicrobesNG Sequencing Facility (www.microbesng.com), using the Nextera XT library preparation kit (Illumina, USA) and sequenced on an Illumina HiSeq/NovaSeq platform (250-bp paired-end reads). The downstream analysis of the sequencing data is ongoing.

Data was generated by Amy Wedley^1^ and Paul Wigley^1,2^

^1^Institute of Infection, Veterinary and Ecological Sciences, University of Liverpool, Liverpool, UK.

^2^School of Veterinary Sciences, University of Bristol, Bristol, UK.

**Table S1: Culture media, growth conditions, and identified bacterial species isolated from chicken caecal contents.**

| **Culture media** | **Culture conditions** | **Number of unique species identified** | **Organisms present** |
| --- | --- | --- | --- |
| Columbia Blood Agar | Aerobic | 10 | *Bacillus altitudinis*  *Bacillus cereus*  *Bacillus pumilus*  *Bacillus subtilis*  *Enterococcus faecium*  *Enterococcus faecalis*  *Escherichia coli*  *Lactobacillus paracesei*  *Solibacillus silvestris*  *Staphylococcus cohnii* |
|  | Microaerobic | 10 | *Bacillus amyloliquefaciens*  *Bacillus amyloliquefaciens_ssp_plantarum*  *Bacillus cereus*  *Bacillus megaterium*  *Bacillus mojavensis*  *Bacillus pumilus*  *Bacillus subtilis*  *Clostridium perfringens*  *Enterococcus faecium*  *Escherichia coli* |
|  | Anaerobic | 2 | *Clostridium perfringens*  *Enterococcus faecium* |
| Reinforced clostridial agar | Anaerobic | 2 | *Enterococcus faecium*  *Lactobacillus reuteri* |
| De Man, Rogosa and Sharpe (MRS) Agar | Anaerobic | 2 | *Lactobacillus crispatus*  *Lactobacillus johnsonii* |

**Reference:**

Pottenger S, Watts A, Wedley A, Jopson S, Darby AC, Wigley P (2023). Timing and delivery route effects of cecal microbiome transplants on Salmonella Typhimurium infections in chickens: potential for in-hatchery delivery of microbial interventions. Animal Microbiome, 5: 11. <https://doi.org/10.1186/s42523-023-00232-0>
