# Supplementary material for: Lactobacilli Isolated from the Caecum of Healthy Broilers with Antimicrobial Activity are Probiotic Candidates for Controlling Salmonella
Source: Probiotics Antimicrob Proteins. 2026 Mar 19;18(6):7723–43. doi: 10.1007/s12602-026-10980-5 (PMC13518464; doi:10.1007/s12602-026-10980-5)
Supplement: Supplementary file 2 [file 12602_2026_10980_MOESM2_ESM.docx]

**Supplementary Information file S****2**

**Lactate dehydrogenase (LDH) cytotoxicity assay of lactobacilli CFCS on 8E11 chick epithelial cells**

**Fig. S2: Cytotoxicity levels in 8E11 chick caecal epithelial cells following treatment with lactobacilli CFCS.**

Lyophilised, reconstituted (10X) CFCS from *Lact. johnsonii*, *Lact. reuteri* and *Lact. crispatus* overnight cultures and 10X filtered MRS culture broth alone, were assessed for 8E11 epithelial cytotoxicity over 3 h using the lactate dehydrogenase (LDH) Cytotoxicity Detection Kit (Roche; Mannheim, Germany), following the manufacturer protocol. Cell culture medium collected post-treatment (100 µL aliquots) were added to wells in a 96-well plate, including fresh cell culture medium as a background control, and the LDH assay reaction conducted at room temperature for 30 min. Absorbance was measured at optical density (OD) 490nm. Results were expressed as a percentage of total maximum releasable intracellular LDH in control untreated cells (100%), following lysis with 1 mL of 1% v/v Triton X-100 for 5 min. All lactobacilli CFCS-treated groups exhibited low or negligible cytotoxicity, with *Lact. reuteri* and *Lact. crispatus* displaying cytoprotective effects. Data is reported as mean ± SD for N=4 independent lactobacilli cultures, assayed in triplicate (n=3).
