# Supplementary material for: Lactobacilli Isolated from the Caecum of Healthy Broilers with Antimicrobial Activity are Probiotic Candidates for Controlling Salmonella
Source: Probiotics Antimicrob Proteins. 2026 Mar 19;18(6):7723–43. doi: 10.1007/s12602-026-10980-5 (PMC13518464; doi:10.1007/s12602-026-10980-5)
Supplement: Supplementary file 3 [file 12602_2026_10980_MOESM3_ESM.docx]

**Supplementary Information file S3**

**Size fractionation of antimicrobial metabolites in lactobacilli CFCS**

**Fig. S3: Size fractionation of antimicrobial metabolites in lactobacilli CFCS using Amicon® ultra centrifugal filters with molecular weight cut-offs.** Lyophilised, reconstituted (10X) CFCS from (a) *Lact. johnsonii*, (b) *Lact. reuteri* (c), *Lact. crispatus* and (d) 10X filtered MRS culture broth alone, were subjected to centrifugal ultrafiltration with Molecular Weight Cutoffs of 3, 10 and 30 kDa, centrifuged at 14,000 x *g* for 10 min (for 30 kDa and 10 kDa devices) or 30 min (3 kDa device) as per manufacturer recommendations. Flow-through filtrate was collected and retained solutes were recovered by reverse centrifugation (1,000 x *g*, for 2 min). Each was made up with sterile water to a final volume of 500 µL. Filtrate (pass through components) and retained components were tested for anti-*Salm*. Typhimurium 4/74 activity by agar diffusion assay and compared to a pre-ultrafiltration 10X CFCS. Data is reported as mean ± SD of N=3 independent experimental cultures. Non-fractionated 10X filtered MRS broth, 10X CFCS from cultured lactobacilli, and 10 µg Gentamicin antibiotic discs were used as inhibition positive controls. kDa, kiloDaltons.
