# Supplementary material for: Lactobacilli Isolated from the Caecum of Healthy Broilers with Antimicrobial Activity are Probiotic Candidates for Controlling Salmonella
Source: Probiotics Antimicrob Proteins. 2026 Mar 19;18(6):7723–43. doi: 10.1007/s12602-026-10980-5 (PMC13518464; doi:10.1007/s12602-026-10980-5)
Supplement: Supplementary file 4 [file 12602_2026_10980_MOESM4_ESM.docx]

**Supplementary Information file S4**

**Size-exclusion chromatography of lactobacilli CFCS and isolation of anti-*Salmonella* active fractions**

**Methods S4:**

To identify the size of active compounds in lactobacilli CFCS, the supernatants were fractionated using Sephadex G25 gel filtration chromatography. Bovine serum albumin (BSA) and phenol red served as molecular weight markers (approximately 66 kDa and 354 Da), eluting in fractions 4 and 8, respectively (see **Fig. S4.1a**). For lactobacilli 10X CFCS (*Lact. johnsonii*, *Lact. reuteri* and *Lact crispatus*), the size fractions of compounds showing high absorbance at 280 nm (A_280nm_), ranging from fractions 2 through to 15, with a peak seen in fractions 7-8, aligning with the elution profile of phenol red. The components within 10X filtered MRS broth were primarily observed in fraction 6 (see **Fig. S4.1b**).

Agar diffusion assay for antimicrobial activity against *Salm.* Typhmurium 4/74, identified inhibitory fractions of CFCS of *Lact johnsonii*, *Lact. reuteri* and *Lact crispatus*, as presented in **Fig. S4.2**. Results revealed that eluted lactobacilli CFCS anti-*Salmonella* activity in was detected in fractions 8 to 10. Inhibitory activity for *Lact johnsonii* and *Lact crispatus* was concentrated in fractions 8 and 9, while *Lact. reuteri* inhibitory fractions were 8 to 10. Fractionation of MRS alone, resulted in no inhibitory activity.

Overall lactobacilli CFCS antimicrobial metabolites appear to be low molecular weight moieties.

**Fig. S4.1:** **Molecular size characterisation of lactobacilli CFCS components/metabolites using Sephadex G25 gel column chromatography.**

Fractionation using Sephadex G25® PD-10 gel column chromatography of (a) bovine serum albumin (~66 kDa; measured at absorbance (A) 280nm (A_280nm_) and phenol red (~364 Da; measured at A_570nm_) and (b) three lactobacilli lyophilised, reconstituted (10X) CFCS (*Lact. johnsonii*, *Lact. reuteri* and *Lact. crispatus*) compared to 10X filtered MRS culture broth alone. Reported as mean ± SD for triplicate experiments. BSA, bovine serum albumin; Da, kiloDaltons.

**Figure S4.2 Assessment of the anti-Salmonella Typhimurium 4/74 inhibitory activity of Sephadex G25 gel column chromatography fractions of lactobacilli CFCS.**

Lyophilised, reconstituted (10X) CFCS from CFCS from (a) *L. johnsonii*, (b) *L. reuteri,* (c) *L. crispatus* and (d) MRS broth alone were fractionated using PD-10 Sephadex G25® gel column chromatography and assessed for anti-*Salmonella* activity in agar diffusion assays. Inhibition zones were measured in mm. 10X CFCS from cultured lactobacilli were used as inhibition controls (Positive control, panels a-c), as well as 10 µg Gentamicin antibiotic discs. Non-fractionated 10X filtered MRS broth served as a positive (non-active) control in panel d. Sterile phosphate-buffered saline (PBS, pH 7.3) was used as the negative control. Data is reported as mean ± SD for triplicate experiments.
