# Supplementary material for: Lactobacilli Isolated from the Caecum of Healthy Broilers with Antimicrobial Activity are Probiotic Candidates for Controlling Salmonella
Source: Probiotics Antimicrob Proteins. 2026 Mar 19;18(6):7723–43. doi: 10.1007/s12602-026-10980-5 (PMC13518464; doi:10.1007/s12602-026-10980-5)
Supplement: Supplementary file 5 [file 12602_2026_10980_MOESM5_ESM.docx]

**Supplementary Information file S5**

**High pressure and high heat tolerance of the antimicrobial activity of lactobacilli CFCS**

**Fig. S5** **Autoclave (high pressure, steam heat) temperatures do not diminish the antimicrobial activity of lactobacilli CFCS.** Lactobacilli cultured overnight under microaerobic conditions, were lyophilised and reconstituted in 1 mL sterile deionised water (10X CFCS). Samples of 10X CFCS from (a) Lact. johnsonii, (b) Lact. reuteri and (c) Lact. crispatus were subjected to autoclave at 121°C, for 20 min, compared to 10X CFCS at room temperature (Untreated CFCS). Samples were tested against Salmonella Typhimurium 4/74 in an agar diffusion assay, with 10 µg Gentamicin antibiotic discs used as inhibition positive controls, and 10X MRS broth alone (N=3).
