# Supplementary material for: Lactobacilli Isolated from the Caecum of Healthy Broilers with Antimicrobial Activity are Probiotic Candidates for Controlling Salmonella
Source: Probiotics Antimicrob Proteins. 2026 Mar 19;18(6):7723–43. doi: 10.1007/s12602-026-10980-5 (PMC13518464; doi:10.1007/s12602-026-10980-5)
Supplement: Supplementary file 6 [file 12602_2026_10980_MOESM6_ESM.docx]

**Supplementary Information file S6**

**The antimicrobial activity of lactobacilli CFCS is unaffected by proteases**

The antimicrobial activity of lactobacilli CFCS against *S.* Typhimurium 4/74 showed no significant differences to that of untreated lactobacilli CFCS following proteolytic enzyme treatment with pepsin (**Fig. S6.1)** or Papain **(Fig. S6.2)**. No bacteriocins/small AMPs appear to be present or bioactive in lactobacilli CFCS (**Fig. S6.3**)

**Fig*.* S6.1** **No observed effect of papain on anti-*Salmonella* activity of lactobacilli CFCS.** Lactobacilli cultured overnight under microaerobic conditions, were lyophilised and reconstituted in 1 mL sterile deionised water (10X CFCS). Samples of 10X CFCS from (a) *Lact. johnsonii*, (b) *Lact. reuteri,* (c) *Lact. crispatus* cultures were subjected to treatment with Papain (1 mg/mL for 3 h, at 37^o^C), then the enzyme heat-inactivated at 90°C for 15 min. The samples were subsequently lyophilised, reconstituted in 1mL sterile deionised water and tested for anti-*Salmonella* Typhimurium 4/74 activity using the agar-well diffusion assay. Samples were compared to heat-inactivated Papain treated CFCS, no enzyme treated CFCS and heat-treated CFCS alone. Gentamicin antibiotic discs (10 µg) were used as an inhibition positive control (N=3). The antimicrobial activity of papain-treated lactobacilli CFCS against *Salm.* Typhimurium 4/74 showed no significant differences to that of untreated lactobacilli CFCS.

**Fig*.* S6.2 No observed effect of pepsin on anti-*Salmonella* activity of lactobacilli CFCS.** Lactobacilli cultured overnight under microaerobic conditions, were lyophilised and reconstituted in 1 mL sterile deionised water (10X CFCS). Samples of 10X CFCS from (a) *Lact. johnsonii*, (b) *Lact. reuteri,* (c) *Lact. crispatus* cultures were subjected to treatment with Pepsin (1 mg/mL for 3 h, at 37^o^C), then the enzyme heat-inactivated at 90°C for 15 min. The samples were subsequently lyophilised, reconstituted in 1mL sterile deionised water and tested for anti-*Salmonella* Typhimurium 4/74 activity using the agar-well diffusion assay. Samples were compared to heat-inactivated Pepsin treated CFCS, no enzyme treated CFCS and heat-treated CFCS alone. Gentamicin antibiotic discs (10 µg) were used as an inhibition positive control (N=3). The antimicrobial activity of pepsin-treated lactobacilli CFCS against *Salm.* Typhimurium 4/74 showed no significant differences to that of untreated lactobacilli CFCS.

**Fig*.* S6.3 No bacteriocins/small AMPs appear to be present or bioactive in lactobacilli CFCS.** Lactobacilli CFCS was subjected to ethyl acetate solvent-based extraction, a common method to concentrate bacteriocins/small anti-microbial peptides (AMPs). Extraction was conducted on overnight (a) MRS broth, and lactobacilli CFCS from (b) *Lact. johnsonii*, (c) *Lact. reuteri* and (d) *Lact. crispatus*. Samples separated into three fractions; an upper solvent ‘extraction’ phase, an emulsion phase and a lower ‘retained’ aqueous phase, excepting CFCS from *L. crispatus* which generated only upper and lower phase fractions, with no emulsion layer evident. Anti-*Salm*. Typhimurium 4/74 activity of fractions was assessed by agar diffusion assay, with 10 µg Gentamicin discs used as an inhibition positive control (N=3).
