# Supplementary material for: Lactobacilli Isolated from the Caecum of Healthy Broilers with Antimicrobial Activity are Probiotic Candidates for Controlling Salmonella
Source: Probiotics Antimicrob Proteins. 2026 Mar 19;18(6):7723–43. doi: 10.1007/s12602-026-10980-5 (PMC13518464; doi:10.1007/s12602-026-10980-5)
Supplement: Supplementary file 7 [file 12602_2026_10980_MOESM7_ESM.docx]

**Supplementary Information file S7**

**The VOC metabolome of CFCS from lactobacilli harbouring anti-*Salmonella* activity**

To classify volatile organic compounds (VOCs) found in the pooled, anti-*Salmonella* active lyophilised, reconstituted CFCS (10X) of *Lact. johnsonii*, *Lact. reuteri* and *Lact. crispatus.* Principal Component Analysis (PCA) revealed that the CFCS of all lactobacilli groups were clearly separated from the MRS broth control and the loading score for PC1 and PC2 are available here in **Table S7.1**. A total of 33 compounds were detected in the CFCS from all three lactobacilli, with 28 identified being found to be significantly different between groups (*p* < 0.05), including 5 organic acids, 7 aldehydes, 9 ketones, 6 pyrazines and 1 aldehyde (see **Table S7.2**).

**Table S7.1 The loading score of PC1 and PC2 of VOC identified within CFCS of lactobacilli overnight cultures demonstrating anti-*Salmonella* activity.**

| Compound | PC1 score | PC2 score |
| --- | --- | --- |
| Butane-2,3-dione | -0.22349 | -0.20562 |
| Butan-2-one | -0.08404 | -0.078417 |
| 3-methylbutanal | -0.20889 | 0.091003 |
| Acetic acid | 0.10999 | -0.20175 |
| 2-methylbutanal | -0.062276 | 0.015341 |
| Pentane-2,3-dione | -0.18405 | 0.19746 |
| Methyl 2-methylprop-2-enoate | 0.17251 | 0.026836 |
| 1-hydroxypropan-2-one | -0.19004 | -0.21069 |
| Pyrazine | -0.040497 | -0.034933 |
| (Methyldisulfanyl)methane | -0.28233 | -0.19191 |
| 2-methylpropanoic acid | 0.18604 | -0.29373 |
| 2-methylpyrazine | -0.022547 | -0.025384 |
| Butanoic acid | 0.18386 | -0.27324 |
| 5-methylhexan-2-one | -0.2013 | -0.20439 |
| 3-methylbutanoic acid | 0.38138 | -0.46699 |
| Heptan-2-one | 0.15615 | 0.23146 |
| Heptanal | -0.14614 | -0.13475 |
| 2,5-dimethylpyrazine | -0.013729 | -0.009051 |
| 2-ethylpyrazine | -0.02646 | -0.031308 |
| 2,3-dimethylpyrazine | -0.015572 | -0.013147 |
| 3-methylsulfanylpropanal | -0.10452 | -0.11466 |
| 2,6-dimethylheptan-3-one | -0.41279 | -0.10395 |
| Benzaldehyde | -0.11071 | -0.16763 |
| 2,3,5-trimethylpyrazine | -0.017272 | -0.022257 |
| Hexanoic acid | 0.18763 | -0.24774 |
| Pyrazine, 2-ethenyl-6-methyl- | -0.029022 | -0.055246 |
| 3-ethyl-2,5-dimethylpyrazine | -0.013555 | -0.020181 |
| 2-phenylacetaldehyde | 0.0069497 | -0.073177 |
| 2-Nonanone | 0.15751 | 0.23397 |
| (E)-5-methyl-2-propan-2-ylhex-2-enal | -0.23314 | -0.17382 |
| Octanoic acid | 0.20764 | -0.19328 |
| Acetone | -0.096836 | -0.068775 |
| 2-methylpropanal | -0.19673 | -0.1898 |

**Table S7.2 Comparison of the relative concentrations of significant VOCs identified using HS-SPME-GC/MS analysis from** **CFCS of *Lact. johnsonii* (T37), *Lact. reuteri* (T42) and *Lact. crispatus* (T43) harbouring anti-*Salmonella* activity, compared to MRS broth.**

| Compound | *p*-value* | FDR* | Significant pairwise comparisons** |
| --- | --- | --- | --- |
| Acids |  |  |  |
| Butanoic acid | 8.48 x 10^-11^ | 4.00 x 10^-10^ | T37-MRS; T42-MRS; T43-MRS; T42-T37; T43-T37 |
| 2-methylpropanoic acid | 8.29 x 10^-10^ | 2.28 x 10^-9^ | T37-MRS; T42-MRS; T43-MRS; T42-T37; T43-T37 |
| Hexanoic acid | 1.58 x 10^-8^ | 3.48 x 10^-8^ | T37-MRS; T42-MRS; T43-MRS |
| Octanoic acid | 1.42 x 10^-7^ | 2.93 x 10^-7^ | T37-MRS; T42-MRS; T43-MRS |
| 3-methylbutanoic acid | 2.22 x 10^-5^ | 3.66 x 10^-5^ | T37-MRS; T42-MRS; T43-MRS |
| Aldehydes |  |  |  |
| 3-methylbutanal | 5.69 x 10^-11^ | 3.13 x 10^-10^ | T37-MRS; T42-MRS; T43-MRS; T42-T37; T43-T37 |
| 2-methylpropanal | 1.08 x 10^-10^ | 4.44 x 10^-10^ | T37-MRS; T42-MRS; T43-MRS; T42-T37; T43-T37; T43-T42 |
| Benzaldehyde | 1.50 x 10^-8^ | 3.48 x 10^-8^ | T37-MRS; T42-T37; T43-T37; T43-T42 |
| 3-methylsulfanylpropanal | 2.96 x 10^-7^ | 5.43 x 10^-7^ | T37-MRS; T42-T37; T43-T37 |
| 2-phenylacetaldehyde | 0.001 | 0.001 | T42-MRS; T43-MRS; T42-T37; T43-T37 |
| (E)-5-methyl-2-propan-2-ylhex-2-enal | 0.001 | 0.001 | T37-MRS; T42-T37 |
| 2-methylbutanal | 0.022 | 0.026 | T37-MRS |
| Ketones |  |  |  |
| Heptan-2-one | 1.63 x 10^-15^ | 5.37 x 10^-14^ | T37-MRS; T42-T37; T43-T37 |
| Nonan-2-one | 3.72 x 10^-13^ | 4.09 x 10^-12^ | T37-MRS; T42-T37; T43-T37 |
| 2,6-dimethylheptan-3-one | 2.14 x 10^-12^ | 1.77 x 10^-11^ | T37-MRS; T42-MRS; T43-MRS; T42-T37; T43-T37; T43-T42 |
| 5-methylhexan-2-one | 2.42 x 10^-11^ | 1.60 x 10^-10^ | T37-MRS; T42-MRS; T43-MRS; T42-T37; T43-T37; T43-T42 |
| Butane-2,3-dione | 2.08 x 10^-10^ | 7.22 x 10^-10^ | T37-MRS; T42-MRS; T43-MRS; T42-T37; T43-T37; T43-T42 |
| Pentane-2,3-dione | 2.19 x 10^-10^ | 7.22 x 10^-10^ | T37-MRS; T42-MRS; T43-MRS |
| 1-hydroxypropan-2-one | 7.09 x 10^-9^ | 1.80 x 10^-8^ | T37-MRS; T42-MRS; T42-T37; T43-T37 |
| Acetone | 2.53 x 10^-7^ | 4.91 x 10^-7^ | T37-MRS; T42-MRS; T43-MRS; T42-T37; T43-T37 |
| Butan-2-one | 7.06 x 10^-7^ | 1.23 x 10^-6^ | T37-MRS; T43-MRS; T42-T37; T43-T37 |
| Pyrazines |  |  |  |
| Pyrazine | 6.0 x 10^-4^ | 9.0 x 10^-4^ | T37-MRS; T42-T37; T43-T37 |
| 2,3-dimethylpyrazine | 0.007 | 0.01 | T37-MRS; T42-T37; T43-T37 |
| 2-methylpyrazine | 7.8 x 10^-3^ | 0.01 | T37-MRS; T42-T37; T43-T37 |
| 2-ethenyl-6-methylpyrazine | 0.011 | 0.014 | T37-MRS; T42-T37; T43-T37 |
| 2,5-dimethylpyrazine | 0.018 | 0.022 | T37-MRS; T42-T37 |
| 2-ethylpyrazine | 0.027 | 0.031 | T37-MRS; T43-T37 |
| Alkanes |  |  |  |
| (Methyldisulfanyl)methane | 4.74 x 10^-10^ | 1.42 x 10^-9^ | T37-MRS; T42-MRS; T43-MRS; T42-T37; T43-T37; T43-T42 |

Footnote: The sample analysis was conducted in triplicate. * Benjamini-Hochberg to control false discovery rate (FDR) was applied to adjust original p-values for multiple comparisons. ** Significance differences at p < 0.05 between groups (e.g. T37-MRS) are indicated and were determined using one-way ANOVA, followed by Tukey’s honestly significant difference (Tukey’s HSD) post-hoc tests. VOC metabolome of 10X CFCS from *Lact. johnsonii* (T37), *Lact. reuteri* (T42) and *Lact. crispatus* (T43), compared to 10X MRS broth alone (N=3 independent cultures for each group).
