# Supplementary material for: Lactobacilli Isolated from the Caecum of Healthy Broilers with Antimicrobial Activity are Probiotic Candidates for Controlling Salmonella
Source: Probiotics Antimicrob Proteins. 2026 Mar 19;18(6):7723–43. doi: 10.1007/s12602-026-10980-5 (PMC13518464; doi:10.1007/s12602-026-10980-5)
Supplement: Supplementary file 8 [file 12602_2026_10980_MOESM8_ESM.docx]

**Supplementary Information file S8**

**The VOC metabolome of Sephadex G25 gel column chromatography fractions of lactobacilli CFCS harbouring anti-*Salmonella* activity**

To classify VOCs found in the pooled, anti-*Salmonella* active Sephadex G25® gel column chromatography fractions from CFCS of *Lact. johnsonii*, *Lact. reuteri* and *Lact. crispatus*, PCA was performed. The PCA results demonstrated that the inhibitory fractions of each lactobacilli CFCS were clearly separated from matched non-inhibitory fractions of MRS broth controls, with *Lact. reuteri* and *Lact. crispatus* positioned closer to each other. The loading plot (**Fig. S8.1a**), featuring PC1 (45.5%) and PC2 (28.8%), explained 74.3% of the total variance, allowing for optimal data separation. The loading score for PC1 and PC2 are available in **Table S8.1**. The differential abundance of the metabolic profiles of inhibitory fractions of lactobacilli CFCS are illustrated in a heatmap (**Fig S8.1b**). In total, 23 out of the 35 metabolites identified (**Table S8.2**) were found to be significantly different between groups (*p* < 0.05). Among the differential metabolites, 6 organic acids, 2 aldehydes, 2 alcohols, 6 ketones, and 7 pyrazines were identified.

**Fig*.* S8.1** **VOC metabolome of the Sephadex G25 gel filtration derived anti-*Salmonella* fractions of lactobacilli CFCS****.** Lactobacilli cultured overnight under microaerobic conditions, were lyophilised and reconstituted in 1 mL sterile deionised water (10X CFCS). CFCS from lactobacilli were each separated by Sephadex G25 gel filtration chromatography, inhibitory (anti-*Salmonella*) fractions identified by agar diffusion assay and pooled for HS-SPME-GC/MS analysis. **(a)** Principal component analysis (PCA) score plot of the VOC metabolome from the fractionated CFCS *Lact. johnsonii* (T37), *Lact. reuteri* (T42) and *Lact. crispatus* (T43), compared to fractionated MRS broth alone (N=3 independent cultures for each group). **(b)** Heatmap illustrating hierarchical clustering analysis of differentially abundant VOCs from the pooled anti-microbial fractions of lactobacilli, identified by HS-SPME-GC/MS analysis. The heatmap colour scale illustrates the relative concentrations of the differential VOC identified. Dark red indicates a relatively high concentration of the compound, with dark blue indicating a relatively low concentration.

**Table S8.1 The loading score of PC1 and PC2 of VOC identified within the pooled gel chromatography generated fractions of lactobacilli CFCS with anti-*Salmonella* activity.**

| Compound | PC1 | PC2 |
| --- | --- | --- |
| Butane-2,3-dione | 0.043822 | 0.19047 |
| Butan-2-one | 0.080001 | 0.2297 |
| 3-methylbutanal | -0.045317 | 0.21123 |
| Acetic acid | 0.16742 | -0.19564 |
| 2-methylbutanal | 0.12728 | 0.064536 |
| Butan-1-ol | 0.23031 | 0.00073124 |
| 2,5-dimethylfuran | -0.077706 | -0.11938 |
| Pentane-2,3-dione | 0.12171 | 0.08093 |
| 1-hydroxypropan-2-one | 0.12267 | 0.27454 |
| Pyrazine | 0.076338 | 0.10902 |
| (Methyldisulfanyl)methane | -0.015173 | 0.026828 |
| 3-methylbutan-1-ol | -0.13171 | -0.20453 |
| 3-methylbut-2-enal | 0.1265 | 0.13363 |
| 2-methylpyrazine | 0.080118 | 0.080037 |
| Butanoic acid | 0.21919 | -0.20938 |
| Heptane-2,3-dione | 0.54781 | 0.08946 |
| 3-methylbutanoic acid | 0.29028 | -0.34305 |
| 2-methylbutanoic acid | 0.25298 | -0.14229 |
| Heptan-2-one | -0.075585 | -0.12055 |
| 2,5-dimethylpyrazine | 0.13384 | 0.090568 |
| 2-ethylpyrazine | 0.11247 | 0.083934 |
| 2,3-dimethylpyrazine | 0.12198 | 0.097232 |
| 3-methylsulfanylpropanal | 0.22814 | -0.26015 |
| 2,6-dimethylheptan-3-one | 0.067538 | 0.24691 |
| Benzaldehyde | 0.11096 | 0.017019 |
| 2-ethyl-6-methylpyrazine | 0.17692 | 0.078512 |
| 2,3,5-trimethylpyrazine | 0.14005 | 0.049588 |
| Hexanoic acid | 0.22607 | -0.21556 |
| Pyrazine, 2-ethenyl-6-methyl- | 0.094638 | -0.019596 |
| 3-ethyl-2,5-dimethylpyrazine | 0.1495 | 0.038185 |
| 2-phenylacetaldehyde | -0.036247 | -0.076119 |
| Octanoic acid | 0.13276 | -0.2235 |
| Acetone | 0.2031 | 0.3555 |
| 2-methylpropanal | 0.025046 | 0.22809 |

**Table S8.2 Comparison of the relative concentrations of significant VOCs identified using HS-SPME-GC/MS analysis in the pooled gel chromatography generated fractions from lactobacilli CFCS harbouring anti-*Salmonella* activity, compared to MRS broth.**

| Compound | *p*-value* | FDR* | Significant pairwise comparisons** |
| --- | --- | --- | --- |
| Acids |  |  |  |
| Butanoic acid | 3.04x10^-7^ | 1.1x10^-5^ | T37-MRS; T42-MRS; T43-MRS; T42-T37; T43-T37 |
| Acetic acid | 2.34 x10^-5^ | 1.40x10^-4^ | T37-MRS; T42-MRS; T43-MRS |
| Hexanoic acid | 1.59x10^-4^ | 5.7x10^-4^ | T37-MRS; T42-MRS; T43-MRS; T42-T37 |
| 3-methylbutanoic acid | 3.93x10^-4^ | 1.18x10^-3^ | T37-MRS; T42-MRS; T43-MRS |
| 2-methylbutanoic acid | 9.01x10^-4^ | 2.03x10^-3^ | T42-MRS; T43-MRS; T43-T37 |
| Octanoic acid | 0.015 | 0.023 | T42-MRS |
| Aldehydes |  |  |  |
| 3-methyl-  sulfanylpropanal | 1.97 x10^-6^ | 2.92x10^-5^ | T37-MRS; T42-MRS; T43-MRS; T42-T37; T43-T37 |
| Benzaldehyde | 5.89x10^-3^ | 0.01 | T42-MRS; T42-T37 |
| Alcohols |  |  |  |
| 3-methylbutan-1-ol | 3.01 x10^-6^ | 2.92x10^-5^ | T37-MRS; T42-T37; T43-T37 |
| butan-1-ol | 0.007 | 0.01 | T43-MRS; T43-T37 |
| Ketones |  |  |  |
| heptane-2,3-dione | 3.24 x10^-6^ | 2.92x10^-5^ | T42-MRS; T43-MRS; T42-T37; T43-T37 |
| 1-hydroxypropan-2-one | 5.60 x10^-5^ | 2.52x10^-4^ | T37-MRS; T42-T37; T43-T37 |
| Acetone | 4.91x10^-4^ | 1.36x10^-3^ | T37-MRS; T42-T37; T43-T37 |
| Butane-2,3-dione | 8.49x10^-4^ | 2.02x10^-3^ | T37-MRS; T42-T37; T43-T37 |
| Pentane-2,3-dione | 1.62x10^-3^ | 3.24x10^-3^ | T42-MRS; T42-T37; T43-T42 |
| 2,6-dimethylheptan-3-one | 5.85x10^-3^ | 0.01 | T37-MRS; T43-T37 |
| Pyrazines |  |  |  |
| 2,5-dimethylpyrazine | 6.13 x10^-6^ | 4.41x10^-5^ | T37-MRS; T42-MRS; T43-MRS; T42-T37; T43-T37 |
| 2,3,5-trimethylpyrazine | 4.11 x10^-5^ | 2.11x10^-4^ | T37-MRS; T42-MRS; T42-T37; T43-T37; T43-T42 |
| 2-ethyl-6-methylpyrazine | 1.52x10^-4^ | 5.71x10^-4^ | T37-MRS; T42-MRS; T42-T37; T43-T37 |
| 3-ethyl-2,5-dimethylpyrazine | 3.5x10^-4^ | 1.14x10^-3^ | T42-MRS; T42-T37; T43-T37; T43-T42 |
| 2-ethylpyrazine | 8.83x10^-4^ | 2.03x10^-3^ | T37-MRS; T42-T37; T43-T37 |
| Pyrazine | 1.04x10^-3^ | 2.2x10-^3^ | T37-MRS; T42-T37; T43-T37 |
| 2-methylpyrazine | 4.7x10^-3^ | 8.9x10^-3^ | T37-MRS; T42-T37; T43-T37 |

Footnote: The sample analysis was conducted in triplicate. * Benjamini-Hochberg to control false discovery rate (FDR) was applied to adjust original p-values for multiple comparisons. ** Significance differences at p < 0.05 between groups (e.g. T37-MRS) are indicated and were determined using one-way ANOVA, followed by Tukey’s honestly significant difference (Tukey’s HSD) post-hoc tests. VOC metabolome of the Sephadex G25 fractionated 10X CFCS from *Lact. johnsonii* (T37), *Lact. reuteri* (T42) or *Lact. crispatus* (T43), compared to fractionated MRS broth (N=3 independent cultures for each group).
